# From Active Compound to Target Epithelium: Local Delivery, Preclinical Model Validity, and Clinical Endpoint Interpretation in Topical Therapy for HPV-Associated Lower Genital Tract Disease—A Narrative Review

**DOI:** 10.3390/jcm15197631

**Published:** 2026-10-02

**Authors:** Sandra Teodora Gavril, Răzvan Popovici, Iustina Petra Solomon Condriuc, Vasile Adrian Sescu, Raluca Haba, Raluca Anamaria Mogoș, Claudia Florida Costea, Alexandru Cărăuleanu

**Affiliations:** 1 “Grigore T. Popa” University of Medicine and Pharmacy, 700115 Iasi, Romaniaraluca.haba@umfiasi.ro (R.H.);; 2 Cuza Voda Hospital, 700038 Iasi, Romania; 3 Department of Ophthalmology, Emergency Clinical Hospital “Prof. Dr. Nicolae Oblu”, 700309 Iasi, Romania

**Keywords:** human papillomavirus, cervical intraepithelial neoplasia, vulvar intraepithelial neoplasia, topical administration, imiquimod, drug delivery, clinical trial endpoints, narrative review

## Abstract

Cervical excision increases the risk of preterm birth, while active surveillance for grade 2 cervical intraepithelial neoplasia offers no active treatment. Topical therapy for human papillomavirus (HPV)-associated lower genital tract disease could address this gap, yet no agent is approved for intraepithelial neoplasia of the cervix, vagina, or vulva, and a 2026 systematic review left open whether cervical treatment reduces progression. Scarcity does not explain this: 52 randomized trials have evaluated adjuvant treatment after excision, but heterogeneity in phenotype, comparator, and endpoint, and incomplete access to primary reports limit their interpretability. Three problems recur. First, the target epithelial population differs by phenotype and mechanism, yet local exposure was seldom characterized, although measurement strategies are set out here. Second, some antiviral claims rely on systems that do not reproduce the differentiation-dependent HPV life cycle. Third, endpoints are read against spontaneous regression approaching the effect sought. Randomized evidence supports imiquimod as a non-surgical option for appropriately selected women with vulvar high-grade squamous intraepithelial lesions; cervical conclusions depend on comparator and endpoint. We propose a conceptual eight-element sequence from clinical intention to inference, an interpretive aid, and not a validated appraisal instrument. Future studies should prespecify these elements and characterize exposure where feasible.

## 1. Introduction

Treating a cervical precancer means removing or destroying part of the transformation zone, and the cost of doing so is measurable in obstetric outcomes. Across 71 studies and more than six million pregnancies, preterm birth before 37 weeks occurred in 10.7% of women treated locally, compared with 5.4% of those untreated (relative risk 1.78, 95% CI 1.60–1.98) [1]. Risk rose with the depth of tissue removed and reached 3.78 (2.65–5.39) in women treated more than once [1]. A subsequent network meta-analysis described the resulting dilemma: more radical excision was associated with lower treatment failure but higher risk of preterm birth, whereas ablative techniques showed the opposite pattern [2]. This trade-off is particularly relevant to patients who may wish to conceive in future.

Gynecology has already responded, although not with a drug. A 2025 consensus statement from the British Society for Colposcopy and Cervical Pathology and the European Society of Gynaecological Oncology records that active surveillance of CIN 2 is now widely practiced, with evidence that 50–60% of these lesions regress without intervention [3]. Meta-analysis of untreated CIN 2 places regression at 50% by 24 months (95% CI 43–57%), and at 60% in women under 30 [4]. In a population-based cohort, active surveillance was not associated with an increased observed risk of noncervical anogenital HPV-related cancer or precancer compared with loop excision [5]. Surveillance avoids immediate treatment, but it does not shorten time to regression or reduce the proportion of lesions that persist, and lesions destined to regress cannot be identified prospectively [3]. Women under surveillance therefore undergo repeated review over an extended period.

These limitations provide a clinical rationale for evaluating non-excisional topical treatment, and no product is approved for this indication. WHO-recommended treatment options for cervical precancer are currently procedure-based [6], and colposcopy capacity limits throughput even in high-income systems [7]. A phase 1 trial of self-administered intravaginal fluorouracil in women living with HIV has tested the proposition directly [8], and acceptability among its participants was reported as high [9]. Two topical agents hold approvals in HPV-associated disease: imiquimod and a standardized green tea catechin ointment, both for external genital and perianal warts [10,11]. Neither is approved for cervical, vaginal, or vulvar intraepithelial neoplasia. Approval boundaries of this kind reflect the evidence package assembled for each indication rather than anatomy alone.

Off-label evidence nevertheless exists at every site, and it does not point uniformly. On the vulva, imiquimod proved non-inferior to surgery in a phase 3 trial, and both imiquimod and cidofovir produced histological responses in a randomized phase 2 comparison [12,13]. On the cervix, comparative evidence favors imiquimod over placebo, whereas excisional treatment achieved higher response rates [14,15], while topical 5-fluorouracil has controlled evidence as primary and as adjuvant treatment [16,17]. Against these sit a single-arm artesunate study reporting regression without a control group [18], and an industry program terminated for lack of efficacy [19]. Section 3 set out this evidence in more detail.

A systematic review published in 2026 searched four databases for randomized trials of cervical topical treatment in women with high-risk HPV and at most CIN 1. It concluded that the available evidence leaves open whether such treatment reduces progression to CIN 2 or worse [20]. Scarcity of trials meeting the population and comparison addressed by that review explains part of that verdict, not all of it, and the wider randomized literature is not uniformly sparse (Section 3). When the background regression rate approaches the effect being sought, an uncontrolled design does not permit the two to be separated. Comparator and outcome selection matter as well: two randomized trials of imiquimod in high-grade cervical disease supported different clinical conclusions, one against placebo with histological regression as its outcome, the other against excision with viral clearance [15,21]. Preclinical support raises a related problem. Claims of antiviral activity may rest on cytotoxic or antiproliferative readouts in HPV-transformed cell lines, which do not reproduce the differentiation-dependent viral life cycle [22,23].

This review takes those difficulties as its subject. For a given topical intervention it asks three questions in sequence. Could the compound plausibly have reached the epithelial population that must be affected in the phenotype under treatment? Could the preclinical model used test the mechanism claimed? Does the reported endpoint support the inference drawn? The components of that sequence are individually well described. The biology of HPV persistence and the identity of the cells that harbor it have been examined in detail, and remain actively debated [22,24,25,26]. The pharmaceutics of cervicovaginal delivery has its own quantitative literature [27]. The trials have been cataloged [28,29] and single agents meta-analyzed [14]. Yet for sinecatechins, an approved topical therapy for external wart disease, the investigators who measured its penetration state that neither the concentration required in tissue nor the duration of exposure needed is known [30]. These literature have different readerships and different conventions, and we did not identify, among the literature reviewed here, a source that integrates their combined consequences for translational inference.

Scope is confined to HPV-associated disease of the female lower genital tract, comprising cervix, vagina, and vulva. Anal and penile disease, oropharyngeal infection, systemic therapy for invasive cervical cancer, and prophylactic vaccination lie outside it and appear only where they clarify the argument. Productive infection, subclinical persistence, condylomatous disease, and transforming lesions are kept distinct throughout, since they neither share a target cell population nor impose the same delivery requirements [22]. Systemically administered therapeutic immunotherapy directed at HPV lies outside this scope as well, although the field has moved during the period reviewed. In August 2025 the US Food and Drug Administration approved zopapogene imadenovec-drba, a non-replicating adenoviral vector expressing a fusion antigen derived from HPV 6 and HPV 11 proteins and given as four subcutaneous injections over twelve weeks, for the treatment of adults with recurrent respiratory papillomatosis [31]. It is relevant here as evidence that HPV-directed immunologic therapy has become approvable, and it falls outside this review on three independent counts: the disease site is the respiratory tract rather than the lower genital tract, the low-risk types addressed are not the high-risk types that drive intraepithelial neoplasia, and the route is systemic, so none of the local delivery constraints developed below applies to it. Nothing in that approval bears on the treatment of cervical, vaginal, or vulvar intraepithelial neoplasia, and therapeutic vaccination is not reviewed here. Within that scope the review has three objectives: (1) to establish, for the principal topical agents, what the biological target is, what tissue exposure the proposed mechanism requires, and whether the exposure achieved has been characterized (Section 4 and Section 5); (2) to determine which preclinical systems can support the mechanistic claims made for these agents and which cannot (Section 6); and (3) to establish how comparator, endpoint, and follow-up constrain the inference available from the trials reported, and to organize these determinations into a single sequence applicable to published and to planned studies (Section 7, Section 8 and Section 10).

## 2. Methods

This is a narrative review. No formal systematic screening, risk-of-bias assessment, or quantitative synthesis was undertaken, and the article should not be read as a systematic review. The review is question-driven rather than enumerative: database searching was used to structure the identification of evidence bearing on four predefined translational domains, and the purpose was translational integration across studies that are ordinarily reported separately, not exhaustive enumeration of the evidence base. No claim of systematic completeness is made, and statements throughout this article about what was or was not identified describe the literature reviewed here rather than the field as a whole.

We searched PubMed/MEDLINE and Scopus from January 2010 to 22 August 2026, combining four conceptual domains. The window opens in January 2010 because the pharmaceutical and preclinical literature this review integrates dates largely from that point: the quantitative characterization of transport through cervicovaginal mucus [27] and the organotypic systems used to evaluate antiviral agents [32] were both reported in 2010, and the randomized trials of topical agents in cervical and vulvar disease followed. Earlier landmark trials and foundational mechanistic reports were retained through the exemption described below, so the restriction bounds the contemporary literature without excluding the foundational work. The first covered HPV and lower genital tract neoplasia, including cervical, vaginal, and vulvar intraepithelial lesions and anogenital warts. The second covered topical, intravaginal, and local administration, together with formulation and drug delivery terms. The third covered preclinical models, including organotypic and three-dimensional tissue culture, pseudovirion systems, and animal models of papillomavirus infection. The fourth covered clinical outcome measures, including histological regression, viral clearance, and recurrence. Landmark trials and foundational mechanistic or methodological publications predating 2010 were exempted from the date restriction and were identified by backward and forward citation chaining from the records retrieved. Current guidelines were consulted directly at their issuing bodies, and trial registries were searched for registered and terminated programs relevant to the agents discussed.

Studies were selected for their relevance to the predefined translational domains addressed in this review, namely target epithelium, local exposure, preclinical model validity, and endpoint interpretation. Non-English full-text reports were not included in the detailed primary-study appraisal; their existence and aggregate findings were considered through English-language evidence syntheses. This restriction is material for one substantial body of randomized evidence, discussed in Section 3. Language of publication therefore determined which reports could be appraised at source, and this language bias is described where it bears on interpretation rather than being left implicit (Section 3 and Section 11). The review was structured with reference to the SANRA quality domains [33].

## 3. Where Topical Therapy Currently Stands

No topical agent is approved for intraepithelial disease of the cervix, vagina, or vulva. Imiquimod and sinecatechins hold approvals for external genital and perianal warts [10,11], and every other use discussed below is off-label. In several instances the off-label evidence is substantial. Evidence for off-label interventions may arise from randomized trials, cohorts, and registries outside a formal registration program, and the distinction between approved and off-label use bears on interpretation as much as on practice.

The agents differ in mechanism, and the difference bears on where each must arrive. The therapeutic effect of imiquimod is attributed principally to TLR7-mediated innate immune activation involving antigen-presenting cells and the downstream responses they initiate [34,35]. Fluorouracil is an antimetabolite acting on cells synthesizing DNA. Cidofovir is a nucleotide analog whose antiviral activity is exerted through the inhibition of viral DNA polymerases; its effects in HPV-associated lesions, which do not encode a viral polymerase, are attributed to actions on host cell proliferation and are not fully characterized. Carrageenan and related polyanions interfere with virion attachment at the epithelial surface and have no established action on intraepithelial disease already present [36]. For sinecatechins, immunomodulatory, antioxidant, and antiproliferative effects have all been proposed, and no single mechanism has been established as responsible for clinical wart clearance. Section 4 develops what each of these requirements implies.

The vulvar literature contains the strongest phase 3 comparative evidence identified in this review. In a phase 3 non-inferiority trial at six Austrian centers, imiquimod matched surgery for vulvar high-grade squamous intraepithelial lesions, with no significant difference in HPV clearance, adverse events, or treatment satisfaction [12]. An earlier randomized phase 2 trial across 32 UK hospitals compared imiquimod with cidofovir under a Fleming design not powered for direct comparison [13]. At a median 18.4 months, women who had responded to cidofovir showed a trend toward longer freedom from disease, which did not reach conventional significance [37]. A Cochrane review of medical interventions for high-grade vulval intraepithelial neoplasia covers the same field [38].

The cervical literature contains more studies, spanning heterogeneous populations, comparators, and outcomes. A randomized trial of self-applied imiquimod suppositories against placebo in CIN 2–3 reported regression, remission, and HPV clearance, all favoring treatment [21]. Two subsequent randomized trials compared imiquimod with excision rather than with placebo, one in Austria [15] and one in Slovenia [39]. Meta-analysis of five studies places the odds ratio at 4.17 (95% CI 2.03–8.54) against placebo and at 14.81 (6.59–33.27) favoring surgery over imiquimod [14]. Available comparative evidence favors imiquimod over placebo, whereas excisional treatment achieved higher response rates. Where a given trial lands between those poles depends on its comparator, its population, and its primary outcome. Table 1 sets out the controlled and comparative evidence.

Fluorouracil has controlled data in two different clinical intentions. As primary treatment of CIN 2 in women aged 18 to 29, eight self-applied doses of 2 g over 16 weeks produced regression in 26 of 28 women against 15 of 27 under observation [16]. As adjuvant therapy after excisional or ablative treatment in 101 women living with HIV, six months of biweekly application reduced any recurrence from 47% to 28% and high-grade recurrence from 31% to 8% [17]. Both trials reported statistically significant benefit for their respective predefined outcomes. Both used 2 g of 5% cream applied intravaginally at intervals of two weeks, differing mainly in the total number of applications and the clinical setting. The available evidence does not establish that either schedule was optimized for its respective intention.

One development program, which begun since the trials above, is described here because its clinical position illustrates the questions this review raises, not because its results are established. ABI-2280 is a prodrug of 9-((2-phosphonylmethoxy)ethyl)guanine diphosphate, which inhibits the human DNA polymerases used by HPV genotypes in viral replication; it is a different parent nucleotide analog from cidofovir, although both belong to the acyclic nucleoside phosphonate class. It is formulated as a self-administered intravaginal insert and is stated to achieve effective cervical concentrations with minimal systemic exposure [40]. Two registered studies define its position. A phase 1/2, open-label, non-randomized dose-escalation study enrolled 44 participants with biopsy-confirmed cervical intraepithelial neoplasia and a visible lesion in its first two cohorts, and biopsy-confirmed p16-positive cervical high-grade squamous intraepithelial lesion without invasion thereafter; its primary outcome was safety and tolerability, and it is recorded as terminated, with the registry giving a sponsor business decision rather than lack of efficacy as the reason [41]. A randomized, double-blind, placebo-controlled study in women aged 25 to 55 with high-risk HPV persisting for at least 12 months and cervical pathology no worse than CIN 1 is recorded in the registry as completed, with 141 participants enrolled [42]. The report of it describes 139 women randomized 3:1 to ABI-2280 or placebo, with a mean duration of infection at baseline of 28.5 months and two expansion cohorts differing in dosing frequency; the primary efficacy endpoint was absence of HPV DNA at 12 weeks for every genotype present at baseline, with two consecutive fully negative results among the secondary endpoints [40]. That report states that high-risk HPV negativity was achieved in nearly half of treated participants at 12 weeks and describes the result as statistically significant [40]; the sponsor has given the corresponding proportions as 46% against 16% under placebo at 12 weeks and 65% against 32% at 24 weeks [43]. This evidence exists as a conference abstract in a journal supplement and in sponsor announcements, so the proportions above are sponsor-reported rather than peer-reviewed: we identified no full peer-reviewed report and no results posted to either registry record, and the program is therefore not entered in Table 1, which reports effect estimates as published. That study is instructive on a further point: it prespecified pharmacokinetic sampling to day 49 and histopathological assessment of a large loop excision specimen at 12 weeks among its secondary outcomes [41], so exposure measurement was planned rather than omitted, and no resulting data have been reported. The agent is investigational: clearance of an investigational new drug application authorizes study, not marketing approval, and no topical agent is approved in cervical disease.

**Figure 1:**
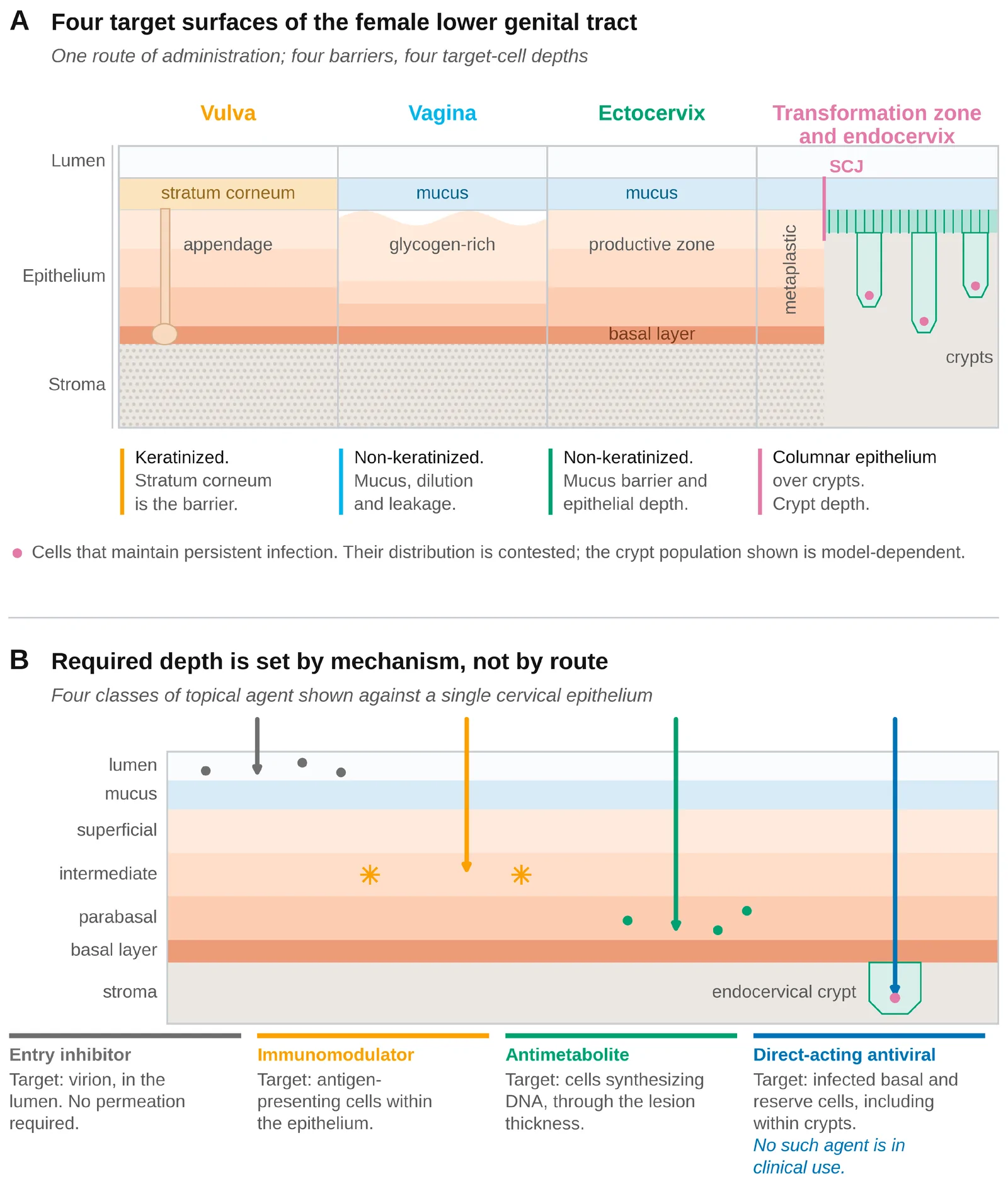
Target epithelium and mechanism-dependent exposure requirements in topical therapy for HPV-associated disease of the female lower genital tract. (**A**) The four anatomical target surfaces reached by local administration, drawn as one continuous epithelial section. The vulva is keratinized, carries a stratum corneum, and contains skin appendages; measured epithelial thickness increases with lesion grade, from 0.31 ± 0.19 mm in uninvolved skin to 0.49 ± 0.23 mm in VIN 1 and 0.69 ± 0.44 mm in high-grade lesions [49]. The vagina and ectocervix are non-keratinized and overlaid by cervicovaginal mucus, in which mean pore diameter is 340 ± 70 nm and steric exclusion alone does not account for the marked restriction of transport observed for certain particles and virions [27]. Product applied to the lumen is subject to dilution, leakage, and limited residence [50]. At the transformation zone, metaplastic squamous epithelium gives way at the squamocolumnar junction to a single columnar layer overlying crypts that extend into stroma. Circles mark cells that maintain persistent infection. Their distribution is contested, having been described both as a discrete junctional population [24] and, under the reserve-cell model, as extending throughout the endocervical epithelium [25,26]; the crypt population shown is accordingly model-dependent and does not represent a defined anatomical boundary. (**B**) The depth a topical agent must reach depends on its mechanism rather than on its route, shown here for a single ectocervical epithelium. An entry inhibitor acts on virions in the lumen and requires no permeation [36]. The therapeutic effect of an immunomodulator such as imiquimod is attributed principally to TLR7-mediated activation of antigen-presenting cells within the epithelium [34,35]. An antimetabolite must reach cells synthesizing DNA throughout the lesion. A hypothetical direct-acting antiviral directed at viral genome maintenance would have to reach infected basal and reserve cells, including those within crypts; the crypt in panel B is an endocervical structure, shown within the same section so that the required depths can be compared, and no such agent is in clinical use. No tissue exposure target has been established for any of these classes: for sinecatechins, an approved topical therapy for external wart disease, the investigators who measured its penetration report that neither the concentration required in tissue nor the duration of exposure needed is known [30]. Approaches by which such exposure could be measured are set out in the Section Operationalizing Local Exposure Measurement in Future Studies Abbreviations: HPV, human papillomavirus; SCJ, squamocolumnar junction; TLR7, Toll-like receptor 7; VIN, vulvar intraepithelial neoplasia. Note: vulvar thickness values are measured [49]; all other proportions are schematic and not to scale, and the depths shown in panel B are conceptual rather than measured.

The program is instructive whether or not its findings are confirmed. Its mechanism places it in the antimetabolite category of Figure 1B: the inhibition of DNA polymerase acts on cells synthesizing DNA, so the exposure required is set by where proliferating infected cells lie rather than by the route of administration. The delivery claim, that the formulation achieves effective cervical concentrations with minimal systemic exposure, appears among the objectives of that report rather than as concentration data within it [40]; it is the claim Section 5 finds to be seldom substantiated by measurement, and although pharmacokinetic sampling was prespecified [41], no cervical tissue concentration has been reported. The virological endpoint, by contrast, is specified to the level this review argues for: negativity is defined against every genotype present at baseline rather than against any high-risk type, and a secondary endpoint requires two consecutive negative results [40], addressing the ambiguity described in Section 7. Its two studies differ in exactly the elements this review treats as decisive: an uncontrolled dose-escalation study in high-grade disease with a safety endpoint, and a placebo-controlled study in persistent infection without high-grade disease with a virological endpoint. The second is not evidence about the first phenotype. What a subsequent phase 2b study can establish will depend on how phenotype, comparator, endpoint, and exposure are aligned in advance, which is the sequence of Section 10.

Two further observations belong in any account of where the field stands. The first is that negative development programs may be incompletely represented in the published literature. A randomized, quadruple-masked phase 2 program evaluating the intravaginal gel 851B across seven dosing regimens spanning three concentrations in cervical high-risk HPV infection was terminated for lack of efficacy, and we identified no full publication of its results; the information available is registry-based [19]. The second is that a substantial parallel evidence base lies outside the trials usually cited in English-language reviews. A Bayesian network meta-analysis of vaginal treatment for persistent high-risk HPV after excisional treatment identified 52 randomized trials in 5460 women across eight interventions; four of those interventions included recombinant human interferon, three of them in combination with a topical preparation [44]. These trials are not appraised individually here. That omission is a limitation of the present work rather than a judgment on the studies, and it should not be read as treating the literature as irrelevant. Several features of the synthesis bear directly on how the evidence base should be described. All 52 trials addressed a single clinical intention, treatment of persistent high-risk HPV after excisional treatment of high-grade disease, and were analyzed against a single virological primary outcome, high-risk HPV clearance, with follow-up of 6 to 24 months. The interventions evaluated were interferon alone, Baofukang, Paiteling, Bletilla striata Sanhuang powder, lactobacilli vaginal capsules, and three interferon-containing combinations, and they overlap only marginally with the agents examined in this review. The synthesis reports 29 of the 52 trials at moderate to high risk of bias, only one trial with more than 100 participants per group, and no effect estimate accompanied by high confidence under GRADE [44]. The relevant point for the present review is therefore not that trials are few. It is that a numerically large randomized literature may still be difficult to apply to a specific translational question when phenotype, treatment setting, comparator, endpoint, follow-up, and intervention composition all differ from it, and when the primary reports cannot be examined at source. Evidence volume and evidence interpretability are distinct quantities, and the argument developed here concerns the second.

**Table 1:** Selected controlled and comparative clinical evidence for topical therapy in HPV-associated disease of the female lower genital tract, with the inference each design supports. Effect estimates are reported as published; where a trial reported arm-level proportions without an accompanying estimate this is recorded as NR, and no estimates were calculated for this table. Trials are grouped by agent rather than by phenotype, and rates are not transferable across phenotypes. Studies of external genital warts are included for the behavior of their control arms and for the parallel evaluation of two formulations of one extract, not as evidence applicable to intraepithelial disease. For reference, cumulative outcomes for untreated CIN 2 at 24 months are regression 50% (95% CI 43–57%), persistence 32% and progression 18%, with regression reaching 60% in women under 30 [4]. The table is selective and is not an inventory of all randomized evidence in this field; the parallel randomized literature on adjuvant treatment after excision described in Section 3 is characterized there and is not reproduced here [44].

Agent	Phenotype	Design and Comparator	n	Outcome, Treatment vs. Control	Effect Estimate	Follow-Up	Supported Inference
Imiquimod 5% [21]	CIN 2–3	RCT, double-blind, self-applied suppositories 16 wk; placebo	59	Histological regression 73% vs. 39%; HPV clearance 60% vs. 14%	NR	16 wk	Effect against placebo. Study biopsies at entry and 8 wk may have contributed in both arms, as the authors note
Imiquimod 5% [15]	Cervical high-grade lesion	RCT, phase 3, open-label, multicenter; LLETZ	93	hrHPV negativity 43.1% vs. 64.3%	Difference 21.2 percentage points (95% CI 0.8–39.1) favoring LLETZ	6 mo	Inferior to excision on the virological endpoint; recurrence not addressed
Imiquimod 5% [14]	Cervical high-grade lesion	Meta-analysis, 5 studies; placebo and surgery	463	Pooled proportion with histological regression to ≤CIN 1: 55% (imiquimod), 29% (placebo), 93% (surgery)	Imiquimod vs. placebo OR 4.17 (95% CI 2.03–8.54); surgery vs. imiquimod OR 14.81 (95% CI 6.59–33.27)	Variable	Favors imiquimod over placebo and surgery over imiquimod, as reported; pooled across heterogeneous endpoints
Imiquimod 5% [12]	Vulvar high-grade lesion	RCT, phase 3, non-inferiority, 6 centers; surgery	98 per protocol	Complete clinical response 80% vs. 79%	Difference in proportion −0.016 (95% CI −0.15 to 0.18); non-inferiority *p* = 0.0056	6 mo	Non-inferior to surgery on clinical response. Invasive disease was found in 5 patients at surgery and in none treated per protocol; excision provides more extensive histological assessment, so this is not evidence that imiquimod prevents invasion
Cidofovir 1% vs. imiquimod 5% [13]	VIN 3 (former ISSVD nomenclature)	RCT, phase 2, open-label, 32 centers; head-to-head	180	Complete histological response 46% vs. 46%	Fleming design, not powered for direct comparison	6 wk after treatment	Both agents active; superiority of neither established
Cidofovir vs. imiquimod [37]	VIN 3, responders only	Follow-up of the above; head-to-head	83	VIN-free at 18 mo 94% (95% CI 78.2–98.5) vs. 71.6% (95% CI 52.0–84.3)	Time to new VIN, imiquimod versus cidofovir: univariable HR 3.46 (95% CI 0.95–12.60), *p* = 0.059; confidence interval spans unity	Median 18.4 mo	Difference in durability not established
Sinecatechins 15% and 10% ointment [45]	External anogenital warts	RCT, double-blind; vehicle	502	Complete clearance 57.2% and 56.3% vs. 33.7%	Both *p* < 0.001	16 wk treatment; 12 wk treatment-free follow-up	Effect against vehicle. Control-arm response does not establish vehicle activity
Polyphenon E 15% ointment and 10% cream [46]	External genital warts	RCT, phase II/III, four-arm, 28 sites; placebo	242	Ointment 15%: 61.0% M, 56.8% F; cream 10%: 53.8% M, 39.5% F; placebo 40.5% M, 34.1% F	*p* = 0.0066 (ointment, sexes combined)	12 wk post-treatment	Two galenic formulations of one extract evaluated in parallel
5-Fluorouracil 5% [16]	CIN 2, primary treatment, age 18–29	RCT; observation	60	Regression 26/28 (93%) vs. 15/27 (56%)	Composite normal outcome RR 2.25 (95% CI 1.05–5.09)	6 mo	Effect at 6 mo; durability not addressed
5-Fluorouracil 5% [17]	Adjuvant after excision or ablation, women with HIV	RCT, phase 3, unmasked, multicenter; observation	101	Any recurrence 28% vs. 47%; high-grade recurrence 8% vs. 31%	*p* = 0.04 (time to CIN); *p* = 0.014 (high-grade)	Up to 18 mo	Effect on recurrence in an immunosuppressed population; background rate differs from immunocompetent cohorts
Sinecatechins ointment [47]	Usual-type VIN	RCT, phase 2, double-blind; placebo	26	No difference in histological response	NR	16 and 32 wk	Negative on the primary histological endpoint; clinical response in all 13 treated participants on a secondary measure. Biopsy before treatment and again at both assessment points; recruitment target not reached, so the trial was underpowered for its primary endpoint
Carrageenan gel [36]	Prevention of incident type-specific HPV infection	Exploratory phase IIB RCT, double-blind, self-applied; placebo gel	461 randomized (429 in incidence analysis)	Acquisition of at least one new HPV type 108/208 (51.9%) vs. 147/221 (66.5%)	HR 0.63 (95% CI 0.49–0.81); *p* = 0.0003	12 months	Reduces incident type-specific infection; addresses a prophylactic intention and not established disease
851B intravaginal gel, 7 regimens [19]	Cervical high-risk HPV infection	Phase 2 RCT, quadruple-masked; placebo. Registry record only; no full publication identified	538	Terminated	NR	NR	Terminated for lack of efficacy. Registry-only evidence; the available information does not distinguish inadequate biological activity from inadequate delivery

## 4. Defining the Therapeutic Target: Disease Phenotype and Target Epithelium

The phrase ‘HPV-associated disease of the lower genital tract’ covers conditions that differ in which cells carry the virus, in what those cells are doing, and in how deep beneath the surface they lie. A condyloma is a hyperproliferative mass of infected epithelium producing virions in its superficial layers. A high-grade intraepithelial lesion is characterized by deregulated E6 and E7 expression and abnormal epithelial differentiation, with reduced virion production, although focal productive infection may coexist. Subclinical persistence is neither: viral genomes are maintained in basal cells at low copy number and low transcriptional output, and the epithelium above them appears normal [22]. Treating these as one disease with one delivery problem obscures differences that determine whether a drug can work.

The productive life cycle is tied to differentiation. Genome amplification, late gene expression, and virion assembly occur in cells that have left the basal layer and are moving toward the surface, while the basal compartment maintains episomes at low copy number [22,23]. Doorbar describes this as a division between an immunologically quiet reservoir compartment and a productive compartment derived from it, and notes that host immunity can suppress the productive stages without necessarily eliminating genomes from infected basal cells [22]. Whole-tissue mapping of two cervices detected HPV in a single tissue set out of 242 in one specimen, indicating that persistent infection can be focal and unevenly distributed [48]. A drug directed at virion production and a drug directed at genome maintenance are therefore aiming at different cells, at different depths within the epithelium.

Which cells maintain persistence at the cervix is contested, and this review does not resolve the dispute. One account, based on immunophenotyping of the squamocolumnar junction, proposes a discrete population of junctional cells implicated in the pathogenesis of cervical cancer [24]. According to a second model, reserve cells extend from the junction throughout the endocervical columnar epithelium to the isthmic border, and high-grade lesions arise from these proliferating reserve cells rather than from a restricted junctional population. The authors of that account report that their observations contradict the junctional hypothesis, and propose that the reserve cell pool may not always be eradicated by a standard loop procedure [25,26]. The evidence identified here does not establish which model is correct. Both nonetheless place the relevant persistent-cell compartments beneath the superficial productive layers, distributed unevenly across the cervix, and in the endocervix potentially within crypts rather than within flat epithelium. These competing accounts are set out here not in order to adjudicate between them, which the evidence identified does not permit, but because each specifies a different compartment for a locally administered agent to reach. A discrete junctional population implies a spatially restricted target at the squamocolumnar junction. A reserve-cell population extending to the isthmic border implies a target distributed along the endocervical canal and into crypts. The required depth, lateral extent, and spatial uniformity of exposure differ accordingly, so an unresolved question about which cells sustain persistence propagates directly into an unresolved specification for local delivery. Until that specification can be stated, no formulation can be shown to have met it, and a negative result in established cervical disease cannot be attributed to the compound rather than to its distribution. The dispute is therefore not incidental to the argument of this review but one of its premises.

Anatomy compounds this. The vulva is keratinized, with a stratum corneum that constitutes a diffusion barrier. Measured epithelial thickness was 0.69 ± 0.44 mm in vulvar high-grade lesions and 0.49 ± 0.23 mm in VIN 1, against 0.31 ± 0.19 mm in uninvolved skin, each involved-versus-uninvolved comparison reaching statistical significance [49]. The vagina and ectocervix are non-keratinized, glycogen-rich, and stratified without a stratum corneum. The endocervix is a single columnar layer overlying crypts that descend into the underlying stroma. Three lower-genital-tract regions therefore encompass four delivery-relevant target surfaces, addressed by local administration under different application geometries and retention conditions, as summarized in Figure 1A.

The consequences differ by drug, because the required tissue exposure depends on the mechanism. A polyanion that blocks virion attachment acts in the lumen and before infection, and needs no permeation [36]. An immunomodulator need not act on the infected keratinocyte directly. The therapeutic effect of imiquimod is attributed principally to TLR7-mediated activation of antigen-presenting cells [34]. Topical application induces migration of Langerhans cells, an intraepithelial population, in cutaneous tissue [35]; direct evidence in genital mucosa is more limited. An antimetabolite such as fluorouracil would, on mechanistic grounds, need to reach cells actively synthesizing DNA within the lesion. This is a requirement derived from mechanism, not a measured exposure specification. A hypothetical direct-acting antiviral aimed at genome maintenance would, on the same reasoning, need intracellular concentrations in basal and, according to the reserve-cell model, reserve cells within endocervical crypts. No such agent is in clinical use, and the category is included here as a design target rather than as an available option. Figure 1B shows these four requirements within a single epithelium.

Evaluating a topical product against a generic notion of epithelial penetration misses the relevant question, which is whether it reaches the population its mechanism requires, in the phenotype and at the site being treated. It also follows that the same compound may be adequately delivered for one indication and inadequately delivered for another. The approved indications of imiquimod and sinecatechins, both confined to external genital and perianal wart disease [10,11], are consistent with that possibility without establishing it, since regulatory scope follows the development program rather than the tissue alone.

## 5. Local Delivery: Can the Active Compound Reach the Relevant Target?

Section 4 established that different mechanisms require exposure at different depths. Whether any topical formulation achieves those exposures is largely uncharacterized, and for most agents in current use, we identified no published attempt to measure them.

One example in which local penetration was directly measured concerns sinecatechins, which are applied three times daily for up to sixteen weeks. Using sequential adhesive tape stripping, investigators followed epigallocatechin gallate concentrations in the stratum corneum after a single application. Concentration fell by roughly 71% at eight hours, 84% at twelve, and 98% at twenty-four, with an estimated half-life of 4.5 h [30]. Concentration also declined with depth within the stratum corneum itself, by about one to one and a half orders of magnitude between the second and the ninth tape strip [30]. The investigators then state the problem plainly: neither the concentration of epigallocatechin gallate required in tissue to induce the intended response, nor the duration for which it must be maintained, is known [30]. This is an approved product, applied to a directly accessible external site. We identified no established tissue exposure target for the other candidates reviewed here. The measurement itself concerns stratum-corneum concentration and does not establish exposure within the viable epithelium.

The same development program offers evidence that the vehicle matters. Two galenic formulations of the same extract were evaluated in parallel in a four-arm randomized trial: an ointment at 15% and a cream at 10%, against placebo [46]. A separate randomized trial in 503 patients compared ointment at 15% and at 10% against vehicle, reporting complete clearance in approximately 53%, 51% and 37% respectively (*p* = 0.01 and *p* = 0.03) [51]. The similar performance of the two ointment concentrations did not demonstrate a clear concentration–response relationship over the range evaluated, while two galenic formulations of the same extract were assessed in parallel. We identified no comparable formulation comparison for any agent applied to the cervix.

For sinecatechins, product labeling reports minimal systemic absorption [11]. Low systemic exposure is reassuring for safety but does not indicate concentration within the epithelium, and the two questions are readily conflated. Systemic absorption after topical imiquimod is likewise described as low [10], although systemic adverse effects occur; a single case report describes telogen effluvium after intravaginal use, resolving over nine months [52]. That observation is anecdotal, compatible with systemic exposure, and carries no pharmacokinetic weight. What remains uncharacterized in either case is the concentration achieved at the intraepithelial antigen-presenting cell population on which the proposed mechanism acts.

Before a compound reaches the epithelium, it must persist in the lumen, and cervicovaginal conditions can reduce or alter sustained local exposure. Applied semisolids are diluted by vaginal fluid, and dilution of 10 to 30% measurably alters the rheological properties that govern how a gel spreads and where it remains [50]. Coating is incomplete and uneven, and product is lost by leakage. Vaginal pH, determined largely by lactobacilli and the lactic acid they produce [53], constrains which compounds remain stable and in which ionization state, and shifts toward neutrality with menopause, dysbiosis, and semen exposure. Menstruation may alter or interrupt dosing in some intravaginal regimens. Each of these bears on how much of an applied dose remains at the epithelial surface after application, and these parameters were rarely reported in the clinical trials reviewed here.

A commonly used vaginal-fluid simulant is the Owen and Katz formulation, whose composition was derived from a review of studies on human vaginal secretions published between 1925 and 1997 [54]. It reproduces pH, osmolarity, and ionic composition, which its authors identified as relevant physicochemical variables for drug release and distribution, and they noted that improved simulants would require further work [54]. It does not reproduce the mucin architecture through which a compound must be transported. Formulation performance measured against this medium may therefore be reproducible without being predictive of behavior in vivo.

Transport through mucus is not primarily a question of pore size. Mean pore diameter in human cervicovaginal mucus is 340 ± 70 nm, with a range extending from roughly 50 to 1800 nm [27]. Herpes simplex virions of approximately 180 nm were nonetheless effectively immobilized in that mucus, diffusing several orders of magnitude more slowly than larger but non-adhesive particles [27]. Steric exclusion alone therefore does not account for the marked restriction of transport observed for certain particles and virions, and adhesive interaction with the mucin network has been implicated. Whether this extends to formulated carriers of anti-HPV compounds has not been established. In one vaginal-mucosa-mimicking permeation model, increased mucoadhesion of liposomal carriers was associated with reduced permeation [55]. Where increased mucoadhesion reduces epithelial permeation, a formulation may be better suited to luminal than to deeper tissue targets; the magnitude and direction of this relationship are likely to be formulation- and compound-dependent.

Anatomy determines what happens once the epithelial surface is reached. For intravaginal administration in particular, a single applied volume may distribute across surfaces with different barriers and different target depths, as set out in Table 2.

These observations do not demonstrate delivery failure. Rather, direct characterization of formulation residence, dilution behavior, stability, or tissue exposure was uncommon in the trials identified. Formulation composition may be reported only at the level of a trade name, and we identified no established target concentration against which a measurement could be compared. A negative trial in these circumstances does not permit an inactive molecule to be distinguished from an inadequately delivered one, and a positive clinical signal does not by itself establish that the proposed mechanism mediated the observed effect.

### Operationalizing Local Exposure Measurement in Future Studies

The preceding sections identify an absence of exposure data rather than an absence of methods for obtaining them. Techniques used routinely in dermatological and vaginal-microbicide development were, with the single exception discussed below, not applied within the topical anti-HPV development programs reviewed here. What follows sets out which of these are established, which remain investigational, and which are constrained by the anatomy of the site or by what can reasonably be asked of a participant. No single method serves every subsite, and the choice follows from whether the compartment the mechanism implicates is luminal, intraepithelial, or subepithelial. The approaches are summarized in Table 3.

Luminal exposure is the most accessible of these measurements and the least invasive. Cervicovaginal fluid can be sampled serially by swab, wick, or lavage, and the compound quantified by a validated liquid chromatography–tandem mass spectrometry assay, yielding a concentration–time profile at prespecified intervals after a single dose and at steady state. This characterizes what remains at the epithelial surface and for how long, and is sufficient on its own only where the mechanism operates in the lumen as an entry inhibitor [36]. Residence and dilution can be characterized in the same samples by recovering the applied formulation against a marker, and stability assessed at measured vaginal pH [50,53]. These measurements apply to the vagina and ectocervix and require no procedure beyond sampling.

Tissue exposure requires sampling the epithelium itself, and here the sites diverge and should not be treated alike. On the vulva, sequential adhesive tape stripping yields a depth-resolved profile through the stratum corneum without a surgical procedure, as it has for epigallocatechin gallate [30]; the method is not transferable to non-keratinized mucosa, and even at the vulva it quantifies the barrier rather than the viable epithelium beneath it. At the vagina and ectocervix, a biopsy taken at a prespecified interval after dosing and quantified by validated mass spectrometry, with spatially resolved imaging where such methods are available, is the only direct route to intraepithelial concentration identified here. Such sampling is defensible where a biopsy is already indicated diagnostically or by the trial’s histological endpoint, and is harder to justify for pharmacokinetic purposes alone; it also removes tissue, which is itself a procedural co-intervention where regression is the outcome measured (Section 7). At the transformation zone and within endocervical crypts, we identified no method capable of establishing concentration at the compartment the reserve-cell model implicates [25,26], and this remains the least tractable measurement in the field.

Where in vivo tissue sampling is not feasible, ex vivo and reconstructed systems supply comparative rather than absolute information. Excised human cervical or vaginal tissue mounted in a diffusion cell, organotypic epithelial constructs, and mucosa-mimicking permeation platforms each allow formulations to be ranked against one another under controlled conditions [55]. None establishes that the resulting exposure is therapeutically sufficient, because no target concentration has been defined [30]. Their value is comparative, and it addresses a question that no clinical study identified in this review has asked: whether one vehicle delivers more of the same compound than another.

Two further measurements bear on exposure without quantifying the compound. A pharmacodynamic biomarker sampled from treated epithelium, such as local induction of the transcriptional response an immunomodulator’s mechanism predicts, can demonstrate target engagement, provided the biomarker has been validated for that mechanism and is kept distinct from the efficacy endpoint. Adherence should be measured rather than assumed, because a self-applied regimen extending over weeks generates variation in delivered exposure that is indistinguishable at analysis from variation in response; applicator return, weight of returned product, and electronic dosing records are each feasible in this setting.

Independent of which of these a given study can support, a minimum reporting set is achievable in any trial without additional intervention burden. Studies should report the active concentration and the dose delivered per application, the formulation rather than a trade name alone, the anatomical site and method of application, the frequency and total duration of dosing, contact or retention time where measurable, adherence and how it was ascertained, and the timing of any biological sample relative to the last dose. Where a local pharmacokinetic or pharmacodynamic measure is feasible, it should be prespecified together with its sampling schedule. Reporting at this level would not by itself establish adequate delivery. It would, however, allow a negative result to be examined for insufficient exposure rather than leaving compound and vehicle equally implicated, which is the position in which the terminated intravaginal gel program leaves the reader [19].

## 6. What Preclinical Models Can, and Cannot, Demonstrate

If delivery has largely not been measured, the models used to justify a mechanism carry more of the interpretive weight. Many commonly used models address only a subset of the biological processes implied by the corresponding mechanistic claim.

These distinct properties are sometimes grouped under the broad description of anti-HPV activity. Cytotoxicity is the capacity to kill cells. Antiproliferative activity is the capacity to slow their division. Inhibition of viral entry is the capacity to prevent virions from establishing infection. For the purposes of this framework, antiviral activity is defined as interference with the viral life cycle in already-infected cells. Activity against productive infection is interference with genome amplification, late gene expression, and virion assembly. Activity against transformed HPV-associated cells is an effect on cells whose phenotype is driven by deregulated E6 and E7 expression but which are no longer producing virus. A compound may possess any one of these without the others.

Common monolayer screening systems address the first two and the last of those properties. HeLa, SiHa, and CaSki are cervical carcinoma lines carrying integrated HPV genomes; they do not stratify, do not support the productive life cycle, and do not produce virions. Demonstrating that a compound reduces their viability establishes a cytotoxic or antiproliferative effect on transformed cells derived from cervical cancer. It does not by itself establish activity against the differentiation-dependent viral life cycle, because that cycle is not proceeding in the culture. The limitation reflects the differentiation dependence of the HPV life cycle: productive virion generation requires a differentiation-competent epithelial system, classically reproduced in organotypic culture [23,32]. This has been known since the original demonstration that HPV biosynthesis follows epithelial differentiation [23], and it remains a central obstacle to conventional antiviral screening in this virus.

Two systems circumvent the requirement for differentiated tissue, and both answer bounded questions. Pseudovirions, assembled from capsid proteins around a reporter plasmid, and quasivirions, which package the viral genome, permit analysis of infectivity, transmission, and neutralization [56]. A compound that blocks pseudovirion entry has demonstrated interference with attachment or internalization. That is the property a prophylactic barrier product requires, and a property that does not directly address infection already established within the epithelium, where acquisition occurred months or years earlier.

Any system used to study the productive cycle must support epithelial differentiation. Organotypic raft culture remains a principal experimental system meeting that requirement, including virion morphogenesis, and has been used to evaluate antiviral agents [32,56,57]. Its limitations are practical rather than conceptual. Throughput is low and the technique demands sustained expertise, so it is rarely used for screening. Not every viral genome performs, either: the prototype HPV16-containing cell line has failed to yield infectious virus in raft culture, while a clinically derived HPV16 genome introduced into primary keratinocytes does [58]. A screening result obtained in this system may therefore depend on which isolate the laboratory maintains. Three-dimensional cervical and oral tissue models extend the organotypic approach to defined host–pathogen readouts [59].

Animal work carries a specific constraint. The MmuPV1 mouse papillomavirus model reproduces papillomavirus-mediated disease of the female reproductive tract and has been productive for studies of persistence and immune control [60]. The standard infection-establishment protocol includes hormonal synchronization followed by nonoxynol-9, which deliberately disrupts the epithelium to potentiate viral entry [60]. That step limits extrapolation of early penetration observations to an intact human mucosal barrier. It does not determine what the model can address in therapeutic experiments conducted after the epithelium has recovered.

None of the biological models discussed above, by itself, evaluates formulation performance. We identified no broadly accepted standardized in vitro release or permeation method for cervicovaginal semisolids, and no consensus simulant beyond the one discussed in Section 5 [54]. Purpose-built tools exist: mucosa-mimicking permeation platforms have been developed and characterized for vaginal formulations [55]. In the literature reviewed here, they have not been applied within anti-HPV development programs, so the biological and pharmaceutical model systems appear to have advanced in parallel.

A system suited to the delivery question would ideally include stratified tissue in which the productive cycle proceeds, a barrier equivalent to the site being treated, and a readout that separates viral events from cytotoxicity. Organotypic culture supplies the first, and animal models can supply the second, subject to the constraint above. Different models may reasonably answer different development questions, and no single universal system is required at every stage. We did not, however, identify an established system integrating all three dimensions. Where none is available, a compound entering clinical evaluation carries an untested assumption about delivery, and a negative trial will be correspondingly harder to interpret.

An early registered trial shows how far molecular specificity can outrun the means of delivering it. In 2017 a single-center study was registered to evaluate transcription activator-like effector nucleases and CRISPR/Cas9 in HPV-related cervical intraepithelial neoplasia grade 1, each nuclease supplied as a plasmid formulated with a poly(β-amino ester) and poloxamer 407 in a gel applied topically twice weekly for four weeks, against an observation arm; the registry describes a phase 1 design with an anticipated enrollment of 60, adverse events as the primary outcome, and HPV16 and HPV18 DNA titers, cytology, and histology as secondary outcomes [61]. The record was last updated in 2017, its recruitment status is given as unknown, no results have been posted, and we identified no peer-reviewed report of it. The registry therefore describes a design rather than a finding, and no clinical conclusion can be drawn from it. The design is nonetheless informative in two respects. An agent directed at viral genomes must reach the interior of infected cells, which is the most demanding version of the exposure requirement set out in Section 4, and a topically applied gel is being asked to accomplish it without any reported measurement of what reaches the epithelium. The endpoint compounds the difficulty: CIN 1 regresses spontaneously at a high rate, so an observation-controlled study of this size would be unlikely to separate a treatment effect from natural history even if the intervention worked, which is the problem set out in Section 7.

Table 4 lists these systems against the inferences each supports. Reduced viability in a cervical carcinoma line does not establish antiviral activity. Inhibition of pseudovirion entry addresses a different clinical intention. Activity in raft culture alone does not establish in vivo delivery, and efficacy in a model whose infection protocol disrupts the epithelium bears only indirectly on crossing an intact one. A statement that antiviral activity has been demonstrated in vitro is therefore insufficient, without specification of the model and the process measured, to predict clinical activity in established intraepithelial disease.

## 7. Clinical Endpoint Interpretation in a Disease with High Background Regression

The outcomes reported in this field are not interchangeable and should not be interpreted as equivalent. Lesion clearance describes what the clinician sees. Cytological regression describes exfoliated cells. Histological regression describes an epithelium sampled at one point by a biopsy that may or may not have struck the site of the diagnostic one. Detection of HPV DNA describes whether viral genome was recovered from the material collected, and answers a different question depending on whether any high-risk type, the originally detected type, or the same type at two separated visits is required. Recurrence and progression to CIN 2 or worse describe events over time, and lie beyond the follow-up most of these trials allowed. Movement in one of these measures does not imply movement in the others.

Whatever measure is chosen carries a background rate, and here the background is large. Half of untreated CIN 2 regresses within 24 months (95% CI 43–57%), with 32% persisting and 18% progressing, and regression reaches 60% in women under 30 [4]. The 2025 BSCCP and ESGO consensus statement puts spontaneous regression at 50–60% and records that active surveillance is now widely practiced on that basis [3]. In an uncontrolled cohort, an observed regression proportion of this magnitude may overlap substantially with the range expected from phenotype-specific natural history. This reasoning applies to uncontrolled designs; it does not invalidate a between-group effect properly estimated in a randomized trial. Comparable phenotype-specific estimates are not available for every lesion grade or site, which constrains what an uncontrolled study can be compared against.

Single-arm studies do not permit the two to be separated. A phase 1 study of intravaginal artesunate reported histological regression in 19 of 28 participants with CIN 2 or CIN 3 [18]. Without a concurrent comparator, the study cannot determine how much of the observed regression was attributable to treatment rather than to natural resolution, particularly since the natural history of CIN 3 should not be assumed to match that of CIN 2.

A concurrent control arm permits estimation of the between-group treatment effect, although substantial response may still occur in the control group. External genital warts are a different phenotype from intraepithelial neoplasia, and the rates that follow are not transferable between them; the trial is informative here for the behavior of its control arm. In the registration trial of sinecatechins, complete clearance occurred in 33.7% of participants receiving vehicle alone, against 57.2% and 56.3% in the two active arms [45]. The between-arm difference is statistically significant in both comparisons. The 33.7% response in the vehicle arm is harder to interpret. It is compatible with natural resolution, with variability between assessors, with regression to the mean in participants enrolled at maximal lesion burden, with behavioral change during a trial requiring three daily applications and repeated clinic attendance, and with an effect of the vehicle itself. These explanations are not mutually exclusive, and the trial was not designed to separate them. Attributing the control response to vehicle activity is a common shortcut that the data do not support.

A further possibility was raised by investigators about their own study. In the randomized comparison of imiquimod with placebo in CIN 2–3, cervical biopsies were taken at enrollment and again at eight weeks. The authors observed that the host’s capacity to clear the lesion may have been favorably influenced by local immune stimulation and by mechanical removal of part of the lesion, and described this as a shortcoming of their design [21]. Placebo-arm regression in that trial was 39% [21]. Where the act of measurement plausibly contributes to the outcome measured, it does so in both arms, so the between-arm difference remains informative while the absolute rates do not. The same consideration applies wherever a trial biopsies a lesion and then measures its regression or clearance, and more than one study reviewed here is affected. The randomized trial of sinecatechins ointment in usual-type vulvar intraepithelial neoplasia obtained a biopsy before treatment and repeat biopsies at both 16 and 32 weeks, a sampling schedule at least as intensive as that of the imiquimod trial, and its report does not discuss any contribution of the procedure to the outcome [47]. Stated as a principle: a diagnostic or protocol-mandated biopsy is a procedural co-intervention whenever regression or clearance is subsequently measured, and should be reported and interpreted as one. This does not imply that biopsy produces a clinically meaningful therapeutic effect in general, and its consequences are not symmetrical between positive and null trials. Where a between-arm difference is reported, a shared procedural effect inflates the absolute rates in both arms without necessarily biasing the difference between them. Where no difference is reported, the same effect operates in both arms and may reduce the difference available to be detected, which carries more weight in a trial that did not reach its recruitment target, as the sinecatechins trial did not [47].

Absence of detectable HPV DNA is not eradication, and the sampling problem has been measured. Two cervices removed for indications unrelated to cervical abnormality were sectioned into 242 and 186 sets respectively, each tested separately; in the first, a single set of 242 was positive for HPV31 [48]. Persistent infection can therefore be focal. Viral genomes may persist focally or at low copy number in basal compartments that a cervical swab or brush samples incompletely, and detection depends on the sampled fraction, the anatomical distribution of infection, viral copy number, assay analytical sensitivity, and the timing of the sample. A negative test at three or six months is evidence that virus was not recovered from the material collected that day. Sustained type-specific negativity over an adequate interval is a stronger claim, and few topical trials have measured it.

Because these measures are not equivalent, the one selected can shape the conclusion. The clearest demonstration comes from within a single protocol. A double-blind randomized phase 2 trial of sinecatechins ointment for usual-type vulvar intraepithelial neoplasia took histological response at 16 and 32 weeks as its primary outcome, under the biopsy schedule described above, and found no difference between arms. All 13 women receiving sinecatechins nonetheless showed a complete or partial clinical response when best clinical response was assessed. The authors summarize the trial as indicating that the ointment may be a treatment for this condition [47]. Across trials the same divergence appears with additional confounding. Two randomized studies of imiquimod in high-grade cervical disease illustrate how interpretation may differ when trials use different comparators and privilege different primary outcomes. One compared imiquimod with placebo and reported histological regression of 73% against 39% [21]. The other compared it with large loop excision, found high-risk HPV negativity in 43.1% against 64.3%, and concluded that excision remains standard [15]. Population, comparator, clinical question, and design differ alongside the endpoint, and no single factor accounts for the divergence.

Population definition operates in the same way. Complete response to imiquimod in vulvar high-grade lesions was 46% in the randomized phase 2 trial that compared it with cidofovir [13] and 80% in the phase 3 trial that compared it with surgery [12]. The two trials used different classification systems, the earlier applying the historical VIN 1–3 scheme rather than the current distinction between vulvar high-grade squamous intraepithelial lesion and differentiated VIN. Differences in cohort composition are one possible contributor to between-study heterogeneity, alongside prior treatment, lesion extent, and other clinical characteristics; they do not by themselves explain the difference in response proportions.

Follow-up length determines what can be said about durability, which is what matters clinically. Recurrence among complete responders in the RT3VIN trial was assessed over a median of 18.4 months, and the difference between agents did not reach conventional significance [37]. Adjuvant intravaginal fluorouracil after excision in women living with HIV reduced high-grade recurrence from 31% to 8% over 18 months [17]. Extended follow-up was required to characterize recurrence beyond the immediate post-treatment period, and a six-month assessment provides limited information on longer-term recurrence.

One consideration applies to any non-excisional strategy. In the phase 3 trial of imiquimod against surgery for vulvar lesions, invasive disease was identified in five patients at primary or secondary surgery and in none of those treated per protocol with imiquimod [12]. The finding should not be interpreted as evidence that imiquimod prevents invasion. Excisional treatment provides more extensive histological assessment, whereas non-excisional management depends on adequate exclusion of occult invasion before treatment and on subsequent surveillance. Ablative treatments recommended by WHO share the specific feature of yielding no excision specimen [6], although they differ from topical therapy in other respects.

## 8. Host and Local-Environment Modifiers

Response to a topical agent is measured against a background rate, and that rate is not the same in every woman. Several modifiers are established well enough to affect how trials should be read.

Age is the clearest. Regression of untreated CIN 2 reaches 60% in women under 30 against 50% overall [4], so two trials enrolling different age distributions may report different control-arm rates for reasons unrelated to treatment. Prior treatment and lesion classification are further clinical covariates that may contribute to between-study heterogeneity, as the divergent vulvar response proportions in Section 7 illustrate [12,13]. Immune status is a third. The adjuvant fluorouracil trial that showed a reduction in high-grade recurrence was conducted entirely in women living with HIV [17], a population for which WHO recommends a distinct management approach [6]. These are prognostic factors and potential sources of heterogeneity; we identified no interaction analyses establishing them as treatment-effect modifiers.

The local environment adds further variation. Vaginal pH is determined largely by lactobacilli and the lactic acid they produce [53], and shifts with menopause, dysbiosis, and semen exposure, altering both the ionization state of an applied compound and the mucus through which it must pass. Ravel and colleagues described several recurring community-state configurations, dominated by *Lactobacillus crispatus*, *L. gasseri*, *L. iners*, or *L. jensenii*, or comprising a heterogeneous group of anaerobes [62]; subsequent work has refined this classification [63]. Cervicovaginal immunology and microbiota have been associated with both HPV infection and its clearance [64]. Whether these associations are causal, and which bacterial features matter, remains unresolved; understanding of the vaginal microbiota is still developing, and findings for individual species have not been consistent across cohorts [63].

Microbiota composition should therefore be treated as hypothesis-generating rather than as an established determinant of treatment response. We identified no trial of topical therapy for HPV-associated disease that stratified treatment response by baseline vaginal microbiota composition, and no direct evidence of a treatment-by-microbiota interaction. The observation that background rates vary by age, prior treatment, immune status, and local environment does not by itself explain the heterogeneity described in Section 7. It identifies variables that trials have not measured, which is a different and more modest claim.

## 9. Discussion

Three findings emerge from the evidence set out above, and they are worth stating together before a framework is proposed. The first concerns what has been established. Controlled evidence of a treatment effect exists for several agents, but it is site-specific rather than general: randomized evidence supports imiquimod as a non-surgical option in appropriately selected women with vulvar high-grade squamous intraepithelial lesions after adequate exclusion of invasion [12], topical fluorouracil has controlled evidence both as primary treatment of CIN 2 and as adjuvant treatment after excision in women living with HIV [16,17], and a polyanion gel reduces incident type-specific infection without addressing established disease [36]. On the cervix the same agent supports different conclusions depending on comparator and endpoint [15,21], and no agent is approved at any of these sites.

The second concerns why the remainder is difficult to interpret, and it is not principally a matter of how many trials exist. The agents differ in the epithelial population their mechanism requires them to reach, and that requirement is set by phenotype and mechanism rather than by route; the exposure achieved was seldom measured in the studies reviewed here, so a negative result cannot separate an inactive compound from an undelivered one. Some mechanistic claims rest on systems in which the differentiation-dependent viral life cycle does not proceed, so they establish cytotoxicity or antiproliferative activity rather than antiviral activity. The endpoints in routine use are measured against a background of spontaneous regression large enough to approach the effects reported, which constrains what an uncontrolled design can show.

The third concerns the shape of the problem. These difficulties are not independent of one another. Which cells a drug must reach depends on the phenotype treated; which preclinical system is informative depends on the mechanism claimed; which comparator is defensible depends on the clinical intention; and which endpoint can support a conclusion depends on all three. A study can therefore be internally valid and still unable to answer the question its authors ask, because the decisions were taken separately. That observation is what the sequence proposed in Section 10 is intended to make explicit, and it is why this review treats target biology, local delivery, preclinical model, comparator, and endpoint as one problem rather than five.

## 10. A Translational Framework for Appraising and Designing Topical HPV Studies

**Figure 2:**
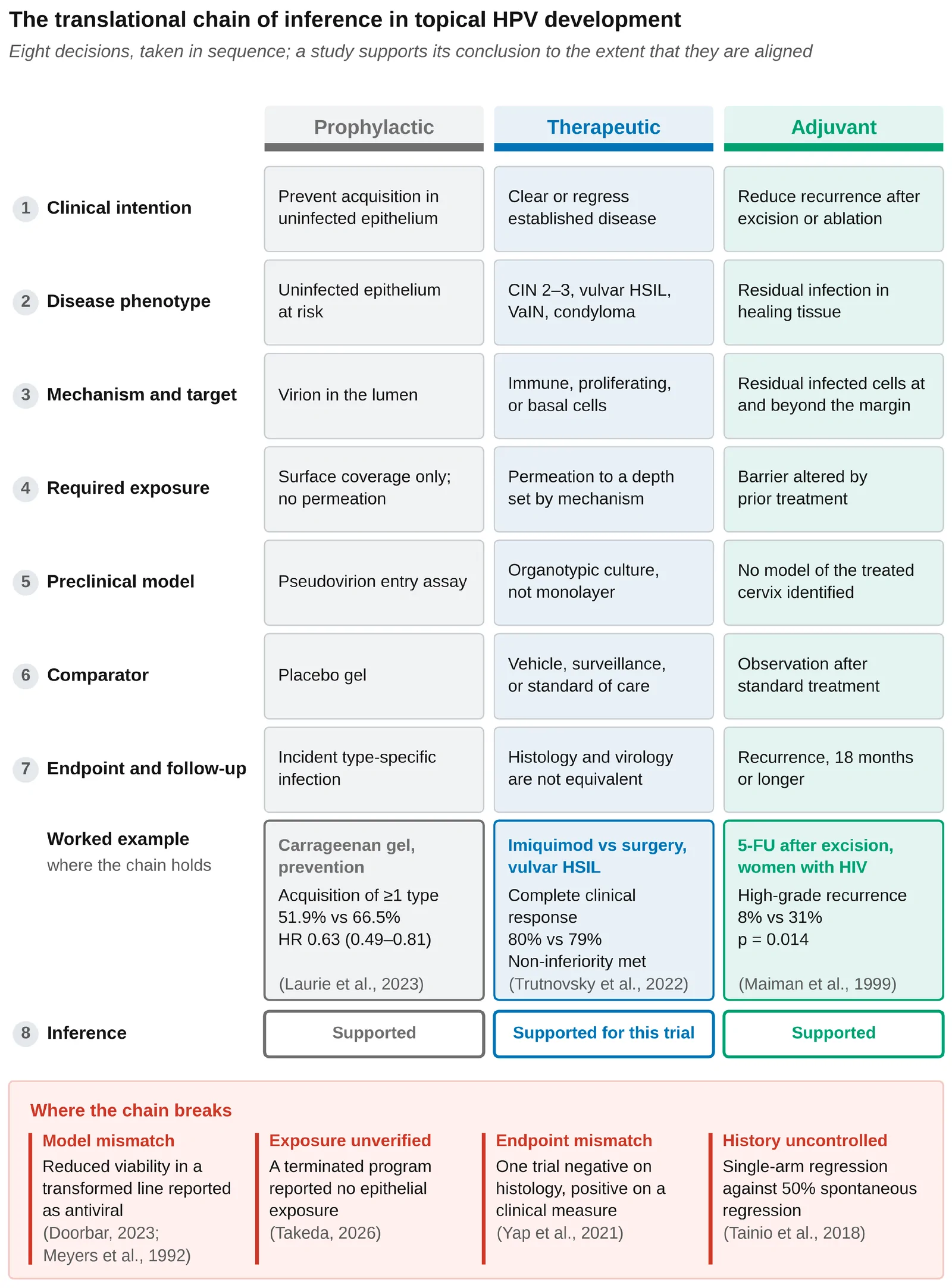
The translational chain of inference in topical HPV development. Eight decisions, taken in sequence, bear on what a study can conclude; alignment across them improves the interpretability of the resulting evidence. The sequence is an author-derived conceptual framework and does not imply that development proceeds in one direction. Rows 1 to 7 show the sequence instantiated for three clinical intentions that share local administration but differ in target, model, comparator, and endpoint. In the prophylactic column an agent acting on virions in the lumen requires no permeation, and an entry assay addresses the intended mechanism directly; the carrageenan trial reduced acquisition of at least one HPV type from 66.5% to 51.9% (hazard ratio 0.63, 95% CI 0.49–0.81) and addresses a prophylactic intention rather than established disease [36]. In the therapeutic column the required depth is set by mechanism rather than by route, and we identified no established tissue exposure target for any candidate [30]; the vulvar non-inferiority trial aligned comparator and clinical endpoint with the treatment intention, reporting complete clinical response in 80% against 79% after surgery [12]. In the adjuvant column treatment has already altered the epithelium; we identified no preclinical model reproducing that post-treatment state, and observation after standard treatment can serve as an appropriate comparator; intravaginal fluorouracil after excision in women living with HIV reduced high-grade recurrence from 31% to 8% over 18 months [17]. Row 8 records the inference each design supports. The lower panel lists four recurring misalignments, each of which can leave a result that is reliable as a measurement, yet unable to answer the question asked: a cytotoxic or antiproliferative readout in a transformed line reported as antiviral activity, in a system in which the differentiation-dependent viral life cycle does not proceed [22,23]; a terminated development program from which no epithelial exposure was reported, leaving both molecule and vehicle equally implicated [19]; a trial negative on its primary histological endpoint and positive on a secondary clinical measure [47]; and single-arm regression that cannot be separated from a background in which half of untreated CIN 2 regresses within 24 months [4]. Effect estimates are reproduced as published, and reference numbers correspond to the reference list of this article. The framework concerns translational interpretability and complements, rather than replaces, conventional assessment of internal validity. Abbreviations: CI, confidence interval; CIN, cervical intraepithelial neoplasia; HPV, human papillomavirus; HR, hazard ratio; HSIL, high-grade squamous intraepithelial lesion; VaIN, vaginal intraepithelial neoplasia; 5-FU, 5-fluorouracil.

The preceding sections describe difficulties that recur independently of the compound under study. We propose that they can be organized as a conceptual sequence of eight decisions, in which the choices made during a study constrain what its results can mean. The sequence is a conceptual synthesis generated in this review rather than an established standard, and it is used for appraisal; it does not imply that translational development proceeds in one direction. Clinical intention defines the clinical question and determines which disease phenotype is appropriate for study. Phenotype and mechanism together determine the target epithelium and the exposure required, and those requirements in turn constrain formulation. Mechanism bears on which preclinical model is informative, while phenotype and intention bear on which comparator and which endpoint, assessed over what interval and with what stratification, can support a conclusion. Alignment across these elements improves the interpretability of the resulting evidence. Where alignment is absent, a result may be reliable as a measurement and still unable to answer the question its authors ask. Figure 2 sets out the sequence and shows it instantiated for three clinical intentions.

The framework concerns translational interpretability. It is intended to complement, not to replace, conventional assessment of internal validity, including randomization, allocation concealment, masking, sample size, adherence, attrition, and outcome ascertainment. It has not undergone formal validation as a risk-of-bias or quality-assessment instrument. It generates no numerical score, assigns no weights to its elements, and is not offered as a substitute for established risk-of-bias instruments; its present status is that of an author-derived conceptual framework for translational interpretation, and it should be read as hypothesis-generating. Formal evaluation would be a separate undertaking. It could proceed by structured expert consensus on the elements and their wording, assessment of content validity against the domains the framework claims to cover, and independent application by more than one appraiser to a prespecified sample of studies, from which agreement between appraisers and concordance with independent expert judgment could be estimated. Comparison against existing appraisal instruments would establish whether the framework identifies interpretive limitations that those instruments do not already capture.

Eight questions follow for a published study. What clinical intention was the product developed for, and does the trial test that intention? Is the phenotype defined, and does the classification system used distinguish HPV-associated from HPV-independent lesions? Is the biological target stated, and is it the population the proposed mechanism acts on? Could the formulation plausibly have delivered the compound to that population, and is anything reported that would allow this to be judged? Does the preclinical evidence cited come from a system in which the claimed mechanism could operate? Was natural history controlled, whether by randomization to a comparator or by a design that accounts for background rates in the population enrolled? Is the endpoint appropriate to the phenotype, assessed over a sufficient interval, and does it correspond to what the authors conclude? And does the evidence presented support the mechanistic claim, or an observation of change?

Published studies may address some elements of the framework and not others. That is not a reason to discard them. It identifies which conclusions are supported and which require additional work, and it distinguishes a study that was uninformative from one that was informative about something other than what it set out to measure.

For a study being designed, the same sequence yields a set of prior decisions. The clinical intention should be defined at the outset. A product intended to prevent acquisition, to treat established disease, or to reduce recurrence after excision faces different biology and different clinical-development requirements, and the trials of fluorouracil in primary and adjuvant use illustrate how far apart these can be [16,17]. The preclinical model should then be selected against the mechanism rather than by convention. For an entry inhibitor, that may mean a pseudovirion assay; for an immunomodulator, a system containing the relevant antigen-presenting cells; and for an agent acting on the productive cycle, an organotypic system.

Formulation should be characterized to the level at which delivery could be judged, which at minimum means reporting composition, and where feasible, residence, dilution behavior, and stability in cervicovaginal conditions. Establishing a tissue exposure target would be more useful still, and we identified no established target for the candidates reviewed here [30]. The measurement approaches by which formulation performance and local exposure could be characterized, their applicability by subsite, and a minimum reporting set are set out in the Section Operationalizing Local Exposure Measurement in Future Studies and Table 3.

Comparator selection should follow clinical and ethical context rather than a single rule. Vehicle-controlled designs remain appropriate where withholding active treatment is acceptable and where the vehicle is characterized. Active surveillance can serve as a comparator for appropriately selected women with CIN 2, because it is an accepted management strategy in that population [3]; it does not extend to CIN 3, to unselected high-grade lesions, or where invasion is suspected. Where treatment should not be deferred, a standard-of-care comparator will generally be appropriate. Inadequate exclusion of occult invasion is a separate matter, bearing on eligibility for non-excisional treatment itself rather than on comparator choice. The vulvar phase 3 trial provides an example in which comparator and clinical endpoint were aligned with the treatment intention [12].

Endpoints should likewise be matched to phenotype and intention rather than standardized across the field. Incident type-specific infection is an appropriate primary virological endpoint for acquisition-prevention studies. For histologically defined intraepithelial disease, histological assessment should generally remain central, although clinical response may also be relevant depending on site and phenotype; histological and virological outcomes should be prespecified according to clinical intention, with discordance between them reported transparently. Recurrence is a clinically relevant endpoint for adjuvant strategies, and follow-up should extend sufficiently beyond treatment to capture it. Stratification variables should be prespecified; age, immune status, and prior treatment are among the clinical covariates most consistently relevant to the populations studied [4,6,12,13,17].

Several of these decisions concern study design and reporting rather than additional intervention burden. Others, particularly exposure characterization and advanced preclinical modeling, require additional resources. What alignment offers is a reduction in interpretive ambiguity. Negative results can arise from an inactive compound, insufficient exposure, an inappropriate target, adherence, population, endpoint choice, bias, or chance, and specifying the chain in advance narrows the range of explanations without identifying the operative one. A structured appraisal framework derived from this section is provided as Table S1.

## 11. Limitations

Study selection was narrative rather than systematic, and no formal screening, duplicate assessment, or risk-of-bias appraisal was performed; relevant work may have been missed. The review accordingly makes no claim to exhaustive coverage of the evidence base, and it cannot support quantitative population-level estimates of its own; the estimates reported here are those of the primary studies and syntheses cited. What it is intended to provide is an integrative reading across the biological, pharmaceutical, and clinical-trial literature that are ordinarily reported and read separately. The review spans three lower-genital-tract anatomical regions, encompassing four delivery-relevant target surfaces, and several disease phenotypes whose natural histories differ, and histopathological nomenclature has changed across the eras from which the cited trials come, so the terminology in the source studies is not uniform. Comparators, endpoints, and follow-up intervals differ across the trials discussed, and we have deliberately avoided pooling or cross-trial numerical comparison for that reason. Much of the delivery argument draws on formulation science developed for other vaginal products, and its extrapolation to HPV therapeutics is indirect. Direct tissue pharmacokinetic measurements are largely absent from the field, so statements about achieved exposure rest on inference rather than measurement. Reports not accessible in English were not appraised at source. This is a language bias, and it is material for the body of randomized evidence described in Section 3. That literature was not disregarded: its intervention set, clinical intention, endpoint, risk-of-bias profile, and certainty ratings were considered through an English-language network meta-analysis [44], and the constraint on the present work is that the primary reports could not be examined individually. Because that literature addresses a single adjuvant intention and a single virological endpoint, its absence from individual appraisal does not bear on the sections of this review concerned with primary treatment or with vulvar disease, but it does limit what can be concluded here about adjuvant treatment after excision. The cell-of-origin and persistence models discussed in Section 4 remain contested, and the delivery argument that follows from them is correspondingly provisional. Finally, the eight-element framework is a conceptual synthesis generated in this review; it has not been prospectively validated and has not been assessed for agreement between appraisers or compared against existing appraisal instruments, and alternative formulations are possible. It should therefore be regarded as an author-derived conceptual framework for translational interpretation rather than as a validated appraisal instrument.

## 12. Conclusions

Topical treatment for HPV-associated disease of the female lower genital tract has produced a substantial body of trials and a smaller body of interpretable evidence. The distance between the two cannot be explained by trial numbers alone, and trials are not uniformly scarce: 52 randomized trials have been identified for adjuvant treatment after excision alone [44]. This arises where the biological target, the delivery achieved, the model used to justify a mechanism, and the endpoint chosen to measure effect have been selected independently of one another.

Reading the literature reviewed here through that sequence changes what can be claimed from it. Several agents have controlled evidence of treatment effects at specific sites, with magnitudes that differ by agent, phenotype, and comparator. Randomized evidence supports imiquimod as a non-surgical treatment option for appropriately selected women with vulvar high-grade squamous intraepithelial lesions after adequate exclusion of invasive disease. Other results, including some of the most encouraging, cannot be separated from natural history in the designs used, and some in vitro claims of antiviral activity rest on systems that do not reproduce the differentiation-dependent viral life cycle. Neither observation implies that the compounds are inactive. Both indicate that features of study design limit what the available results can establish.

For a field now positioned against active surveillance as well as against surgery, that distinction carries clinical weight. Demonstrating benefit will require trials that show an effect beyond phenotype-specific natural history, using appropriately matched comparators, prespecified stratification, and designs in which the proposed mechanism is interpretable. Where it is feasible, a prespecified measure of the exposure actually achieved should be included, since without one a negative result cannot distinguish an inactive compound from an undelivered one (Section Operationalizing Local Exposure Measurement in Future Studies). Defining the clinical intention at the outset is therefore necessary to align biological target, delivery strategy, comparator, and endpoint.

## Figures and Tables

**Table 2:** Anatomical and biopharmaceutical determinants of local delivery across the female lower genital tract.

Subsite	Epithelial Characteristics	Dominant Barrier	Exposure Required, by Mechanism	Formulation Implication	Clinical Consequence
Vulva	Keratinized stratified squamous with stratum corneum; skin appendages present. Measured thickness 0.31 ± 0.19 mm uninvolved, 0.49 ± 0.23 mm in VIN 1, 0.69 ± 0.44 mm in high-grade lesions [49]	Stratum corneum	Immunomodulator: intraepithelial antigen-presenting cells [34,35]. Antimetabolite: proliferating cells through lesion thickness	Retention on an external, directly accessible surface; the region discussed here for which approved topical therapies exist, with approvals covering external wart disease rather than intraepithelial neoplasia [10,11]	The region for which the strongest phase 3 comparative evidence identified in this review exists; imiquimod non-inferior to surgery for high-grade disease [12]
Vagina	Non-keratinized stratified squamous, glycogen-rich, no stratum corneum; rugated; thickness varies with cycle, hormonal status and progestin exposure	Overlying mucus; dilution and leakage	As for ectocervix; agent-dependent	Applied semisolid diluted by vaginal fluid, with 10–30% dilution altering rheology and coating flow [50]; pH determined by lactobacilli and lactic acid [53]	Delivered epithelial exposure was not reported in the clinical trials identified in this review
Ectocervix	Non-keratinized stratified squamous. Productive compartment superficial; basal keratinocytes may maintain low-copy episomes with limited transcriptional activity [22]	Overlying mucus; epithelial thickness	Entry inhibitor: lumen only [36]. Immunomodulator: intraepithelial antigen-presenting cell compartment. Hypothetical direct-acting antiviral: basal layer	Mucus obstructs by adhesion rather than size exclusion; mean pore 340 ± 70 nm, range ≈ 50–1800 nm [27]. In one vaginal-mucosa-mimicking model, mucoadhesive coating was associated with reduced permeation [55]	Where increased mucoadhesion reduces epithelial permeation, it may favor luminal over deeper tissue targets
Transformation zone and endocervix	Metaplastic squamous giving way at the squamocolumnar junction to a single columnar layer over crypts extending into stroma. Reserve cell distribution contested [24,25,26]	Crypt depth; columnar epithelium over stroma	Hypothetical direct-acting antiviral: infected basal and, under the reserve-cell model, reserve cells within crypts. No clinically available agent reviewed here has established exposure at this compartment	Diffusion path may exceed what surface architecture suggests; we identified no formulation characterized specifically against this requirement	According to the reserve-cell model, the site at which excision may leave residual reserve cells [26]; and a particularly challenging site for topical delivery to deeper target compartments

Applies to all subsites. We identified no established tissue concentration target for the anti-HPV candidates reviewed here. For sinecatechins, an approved topical therapy for external genital and perianal wart disease, epigallocatechin gallate in the stratum corneum fell by approximately 71% at 8 h, 84% at 12 h and 98% at 24 h after a single application, with an estimated half-life of 4.5 h, and the investigators state that neither the concentration required in tissue nor the duration needed is known [30]. Abbreviations: HPV, human papillomavirus; VIN, vulvar intraepithelial neoplasia. Notes: Epithelial thickness values for the vulva are measured [49]; all exposure requirements in this table are conceptual, derived from mechanism, and are not measured specifications. Formulation and clinical-consequence entries are interpretive.

**Table 3:** Approaches to measuring local exposure in topical therapy for HPV-associated disease of the female lower genital tract, with what each establishes and its principal limitation. Applicability is stated by subsite; no single approach addresses all four target surfaces described in Section 4.

Domain	Measurement Strategy	What It Establishes	Principal Limitation
Luminal exposure (vagina, ectocervix)	Serial cervicovaginal fluid sampling by swab, wick, or lavage with validated LC-MS/MS; concentration–time profile after a single dose and at steady state	Concentration retained at the epithelial surface and how long it persists; sufficient alone only where the mechanism acts in the lumen [36]	Gives no information on concentration within the epithelium, and sampling itself may remove applied product. Established for other vaginal products
Residence, dilution, and stability (vagina, ectocervix)	Recovery of the applied formulation against a marker in the same samples; rheological assessment after dilution; stability at measured vaginal pH	Whether the product remains at the site of application, and how dilution alters spreading and coating [50,53]	The simulant in general use reproduces pH, osmolarity, and ionic composition but not mucin architecture [54]. Established
Barrier exposure (vulva only)	Sequential adhesive tape stripping with analytical quantification, as reported for epigallocatechin gallate [30]	Depth-resolved concentration within the stratum corneum and its decline over time	Not transferable to non-keratinized mucosa, and quantifies the barrier rather than the viable epithelium beneath it. Established at keratinized sites
Intraepithelial exposure (vulva, vagina, ectocervix)	Biopsy at a prespecified interval after dosing, quantified by validated mass spectrometry, with spatially resolved imaging where available	Concentration actually achieved within the target epithelium, and its variation with depth	Invasive, and defensible mainly where a biopsy is already indicated. The biopsy is itself a procedural co-intervention where regression is an outcome (Section 7). Investigational for this indication
Crypt and reserve-cell compartment (transformation zone, endocervix)	No method identified	—	The compartment implicated under the reserve-cell model [25,26] was not accessible to any approach identified here; the least tractable measurement in the field
Comparative permeation (all subsites)	Ex vivo human tissue in diffusion cells; organotypic epithelial constructs; mucosa-mimicking permeation platforms [55]	Ranking of formulations against one another under controlled conditions	Cannot establish therapeutic sufficiency, since no target tissue concentration has been defined [30]. Established as a pharmaceutical method but not applied in this field
Target engagement (site-specific)	Prespecified, mechanism-matched pharmacodynamic biomarker sampled from treated epithelium	That the compound reached and acted upon the cell population its mechanism implicates	Requires a biomarker validated for that mechanism, and must be kept distinct from the efficacy endpoint. Investigational
Delivered dose and adherence (all subsites)	Applicator count and return, weight of returned product, electronic dosing records	Exposure received rather than exposure prescribed	Measures administration, not tissue concentration. Established

Note: Classification as established or investigational refers to the standing of each method in pharmaceutical and dermatological development generally. Within the topical anti-HPV literature reviewed here, the only such measurement we identified was stratum-corneum tape stripping for sinecatechins in external wart disease [30]; we identified no study of intraepithelial neoplasia reporting any of these measures. Abbreviations: HPV, human papillomavirus; LC-MS/MS, liquid chromatography–tandem mass spectrometry.

**Table 4:** Preclinical models relevant to mechanistic claims in topical anti-HPV development, and the inferences each supports.

Model	Biological Process Represented	Question It Can Answer	Question It Cannot Answer	Inference Not Supported by This Model Alone
HPV-transformed monolayer lines (HeLa, SiHa, CaSki)	Cells carrying integrated HPV genomes with deregulated E6 and E7 expression; no stratification, no productive cycle	Does the compound reduce viability or proliferation of transformed cervical cells?	Anything about viral replication, since the productive cycle is tied to epithelial differentiation and does not occur in monolayer [23]	That a cytotoxic or antiproliferative effect in transformed cells constitutes activity against the viral life cycle
Pseudovirion and quasivirion systems	Capsid-mediated attachment and internalization; reporter or packaged genome	Does the compound interfere with virion entry?	Whether the compound affects infection already established months or years earlier	That inhibition of entry predicts efficacy in intraepithelial disease
Organotypic (raft) culture	Differentiation-dependent stages of the complete life cycle, including virion morphogenesis; used to evaluate antiviral agents [32]	Does the compound act on the productive cycle in stratified tissue?	Whether a formulation delivers the compound to equivalent tissue in vivo	That activity in raft culture alone establishes in vivo delivery. Genome dependence is also relevant: the prototype HPV16-containing line has failed to yield infectious virus in raft culture, whereas a clinically derived HPV16 genome does [58]
Three-dimensional cervical and oral tissue models	Stratified tissue architecture with defined host–pathogen readouts [59]	Host–pathogen interaction in a tissue-like context	Systemic and immune contributions absent from the construct	That a tissue-equivalent result substitutes for an in vivo delivery study
MmuPV1 murine cervicovaginal model	Papillomavirus-mediated disease of the female reproductive tract in vivo [60]	Mechanism, immune response, lesion behavior and persistence	Extrapolation of early penetration observations to an intact human mucosa, since the infection-establishment protocol deliberately disrupts the epithelium with nonoxynol-9 [60]	That efficacy in this model indicates adequate delivery in an untreated human epithelium
In vitro release and permeation systems	Transport of compound across a mucosa-mimicking barrier; formulation-dependent, with mucoadhesive coating reducing permeation [55]	Comparative release and permeation between formulations	Whether the resulting exposure is therapeutically sufficient, since we identified no target concentration [30]	That a permeation result alone establishes adequacy of delivery

Note: Model capabilities and limitations in columns two to four are drawn from the cited sources; the final column is the authors’ interpretation of what each model cannot establish on its own. Six distinct properties are sometimes grouped under the single description of anti-HPV activity: cytotoxicity, antiproliferative activity, inhibition of viral entry, interference with the viral life cycle in infected cells, activity against productive infection, and activity against transformed HPV-associated cells. We identified no broadly accepted standardized in vitro release or permeation method for cervicovaginal semisolids, and the fluid simulant in general use was derived from a review of studies published between 1925 and 1997 [54]. Abbreviations: HPV, human papillomavirus; MmuPV1, Mus musculus papillomavirus 1.

## Data Availability

No new data were created or analyzed in this study. All data discussed are available in the cited publications.

## Supplementary Materials

The following supporting information can be downloaded at https://www.mdpi.com/article/10.3390/jcm15197631/s1. Table S1: Framework for appraising published studies and specifying new ones in topical therapy for HPV-associated disease of the female lower genital tract.

## Abbreviations

The following abbreviations are used in this manuscript:

CI	confidence interval
CIN	cervical intraepithelial neoplasia
HPV	human papillomavirus
hrHPV	high-risk human papillomavirus
HR	hazard ratio
HSIL	high-grade squamous intraepithelial lesion
LLETZ	large loop excision of the transformation zone
OR	odds ratio
RCT	randomized controlled trial
RR	relative risk
SANRA	Scale for the Assessment of Narrative Review Articles
TLR7	Toll-like receptor 7
VaIN	vaginal intraepithelial neoplasia
VIN	vulvar intraepithelial neoplasia

## References

[1] Kyrgiou M., Athanasiou A., Paraskevaidi M., Mitra A., Kalliala I., Martin-Hirsch P., Arbyn M., Bennett P., Paraskevaidis E. (2016). Adverse obstetric outcomes after local treatment for cervical preinvasive and early invasive disease according to cone depth: Systematic review and meta-analysis. BMJ.

[2] Athanasiou A., Veroniki A.A., Efthimiou O., Kalliala I., Naci H., Bowden S., Paraskevaidi M., Arbyn M., Lyons D., Martin-Hirsch P. (2022). Comparative effectiveness and risk of preterm birth of local treatments for cervical intraepithelial neoplasia and stage IA1 cervical cancer: A systematic review and network meta-analysis. Lancet Oncol..

[3] Kyrgiou M., Bowden S.J., Ellis L.B., Hammer A., Lyons D., Freeman-Wang T., Kechagias K.S., Kalliala I., Preti M., Kesic V. (2025). Active surveillance of cervical intraepithelial neoplasia grade 2: 2025 BSCCP and ESGO consensus statement. Lancet Oncol..

[4] Tainio K., Athanasiou A., Tikkinen K.A.O., Aaltonen R., Cárdenas J., Glazer-Livson S., Jakobsson M., Joronen K., Kiviharju M., Louvanto K. (2018). Clinical course of untreated cervical intraepithelial neoplasia grade 2 under active surveillance: Systematic review and meta-analysis. BMJ.

[5] Eriksen D.O., Lycke K.D., Kahlert J., Ostenfeld E.B., Jensen P.T., Wentzensen N., Clarke M.A., Hammer A. (2025). Active surveillance of cervical intraepithelial neoplasia grade 2 is not associated with an increased risk of noncervical anogenital human papillomavirus-related cancer and precancer: A population-based cohort study. Am. J. Obstet. Gynecol..

[6] World Health Organization (2021). WHO Guideline for Screening and Treatment of Cervical Pre-Cancer Lesions for Cervical Cancer Prevention.

[7] Perkins R.B., Wentzensen N., Guido R.S., Schiffman M. (2023). Cervical cancer screening: A review. JAMA.

[8] Adjuvant Self-Administered Intravaginal 5-Fluorouracil Following Cervical Intraepithelial Neoplasia Treatment Among HIV-Positive Women in East Africa. ClinicalTrials.gov Identifier NCT05362955. NCT05362955.

[9] Adewumi K., Kachoria A.G., Adoyo E., Rop M., Owaya A., Tang J.H., Rahangdale L., Mungo C. (2025). Women’s experiences and acceptability of self-administered, home delivered, intravaginal 5-fluorouracil cream for cervical precancer treatment in Kenya. Front. Reprod. Health.

[10] U.S. Food and Drug Administration Imiquimod Cream, 5%: Highlights of Prescribing Information. https://www.accessdata.fda.gov/scripts/cder/daf/.

[11] U.S. Food and Drug Administration (2006). Veregen (Sinecatechins) Ointment, 15%.

[12] Trutnovsky G., Reich O., Joura E.A., Holter M., Ciresa-König A., Widschwendter A., Schauer C., Bogner G., Jan Z., Boandl A. (2022). Topical imiquimod versus surgery for vulvar intraepithelial neoplasia: A multicentre, randomised, phase 3, non-inferiority trial. Lancet.

[13] Tristram A., Hurt C.N., Madden T., Powell N., Man S., Hibbitts S., Dutton P., Jones S., Nordin A.J., Naik R. (2014). Activity, safety, and feasibility of cidofovir and imiquimod for treatment of vulval intraepithelial neoplasia (RT3VIN): A multicentre, open-label, randomised, phase 2 trial. Lancet Oncol..

[14] van de Sande A.J.M., Kengsakul M., Koeneman M.M., Jozwiak M., Gerestein C.G., Kruse A.J., van Esch E.M.G., de Vos van Steenwijk P.J., Muntinga C.L.P., Bramer W.M. (2024). The efficacy of topical imiquimod in high-grade cervical intraepithelial neoplasia: A systematic review and meta-analysis. Int. J. Gynecol. Obstet..

[15] Polterauer S., Reich O., Widschwendter A., Hadjari L., Bogner G., Reinthaller A., Joura E., Trutnovsky G., Ciresa-König A., Ackermann J. (2022). Topical imiquimod compared with conization to treat cervical high-grade squamous intraepithelial lesions: Multicenter, randomized controlled trial. Gynecol. Oncol..

[16] Rahangdale L., Lippmann Q.K., Garcia K., Budwit D., Smith J.S., van Le L. (2014). Topical 5-fluorouracil for treatment of cervical intraepithelial neoplasia 2: A randomized controlled trial. Am. J. Obstet. Gynecol..

[17] Maiman M., Watts D.H., Andersen J., Clax P., Merino M., Kendall M.A. (1999). Vaginal 5-fluorouracil for high-grade cervical dysplasia in human immunodeficiency virus infection: A randomized trial. Obstet. Gynecol..

[18] Trimble C.L., Levinson K., Maldonado L., Donovan M.J., Clark K.T., Fu J., Shay M.E., Sauter M.E., Sanchez K.V., Schwarz F.A. (2020). A first-in-human proof-of-concept trial of intravaginal artesunate to treat cervical intraepithelial neoplasia 2/3 (CIN2/3). Gynecol. Oncol..

[19] Takeda A Study of 851B Gel in Adult Females with Cervical Human Papillomavirus Infection. ClinicalTrials.gov Identifier NCT00312286. NCT00312286.

[20] Pareja R., Agustí N., Vieira S., Mora-Soto N., Sanchez E., Fernandes A., Aristizábal B., Heredia F., Levin G., Varon M.L. (2026). Efficacy of topical treatments for high-risk human papillomavirus in preventing CIN II+ lesions: A systematic review. Int. J. Gynecol. Cancer.

[21] Grimm C., Polterauer S., Natter C., Rahhal J., Hefler L., Tempfer C.B., Heinze G., Stary G., Reinthaller A., Speiser P. (2012). Treatment of cervical intraepithelial neoplasia with topical imiquimod: A randomized controlled trial. Obstet. Gynecol..

[22] Doorbar J. (2023). The human papillomavirus twilight zone: Latency, immune control and subclinical infection. Tumour Virus Res..

[23] Meyers C., Frattini M.G., Hudson J.B., Laimins L.A. (1992). Biosynthesis of human papillomavirus from a continuous cell line upon epithelial differentiation. Science.

[24] Herfs M., Yamamoto Y., Laury A., Wang X., Nucci M.R., McLaughlin-Drubin M.E., Münger K., Feldman S., McKeon F.D., Xian W. (2012). A discrete population of squamocolumnar junction cells implicated in the pathogenesis of cervical cancer. Proc. Natl. Acad. Sci. USA.

[25] Regauer S., Reich O., Kashofer K. (2022). Cervical precancers originate from infected proliferating reserve cells: A comparative histologic and genetic study of thin and thick high-grade squamous intraepithelial lesions. Am. J. Surg. Pathol..

[26] Reich O., Regauer S. (2023). Elimination of reserve cells for prevention of HPV-associated cervical cancer. Virus Res..

[27] Lai S.K., Wang Y.Y., Hida K., Cone R., Hanes J. (2010). Nanoparticles reveal that human cervicovaginal mucus is riddled with pores larger than viruses. Proc. Natl. Acad. Sci. USA.

[28] Desravines N., Miele K., Carlson R., Chibwesha C., Rahangdale L. (2020). Topical therapies for the treatment of cervical intraepithelial neoplasia (CIN) 2–3: A narrative review. Gynecol. Oncol. Rep..

[29] Khamjan N.A., Beigh S., Algaissi A., Megha K., Lohani M., Darraj M., Kamli N., Madkhali F., Dar S.A. (2023). Natural and synthetic drugs and formulations for intravaginal HPV clearance. J. Infect. Public Health.

[30] de Vries H.J.C., Soltanipoor M., Kezic S., Vergunst C.E. (2018). Sinecatechins ointment 10% (Veregen) for genital warts: Percutaneous penetration of epigallocatechin gallate concentrations in the stratum corneum collected by adhesive tape stripping method. J. Eur. Acad. Dermatol. Venereol..

[31] U.S. Food and Drug Administration (2025). PAPZIMEOS (Zopapogene Imadenovec-Drba).

[32] Andrei G., Duraffour S., Van den Oord J., Snoeck R. (2010). Epithelial raft cultures for investigations of virus growth, pathogenesis and efficacy of antiviral agents. Antivir. Res..

[33] Baethge C., Goldbeck-Wood S., Mertens S. (2019). SANRA: A scale for the quality assessment of narrative review articles. Res. Integr. Peer Rev..

[34] Hemmi H., Kaisho T., Takeuchi O., Sato S., Sanjo H., Hoshino K., Horiuchi T., Tomizawa H., Takeda K., Akira S. (2002). Small anti-viral compounds activate immune cells via the TLR7 MyD88-dependent signaling pathway. Nat. Immunol..

[35] Suzuki H., Wang B., Shivji G.M., Toto P., Amerio P., Tomai M.A., Miller R.L., Sauder D.N. (2000). Imiquimod, a topical immune response modifier, induces migration of Langerhans cells. J. Investig. Dermatol..

[36] Laurie C., El-Zein M., Botting-Provost S., Tota J.E., Tellier P.-P., Coutlée F., Burchell A.N., Franco E.L. (2023). Efficacy and safety of a self-applied carrageenan-based gel to prevent human papillomavirus infection in sexually active young women (CATCH study): An exploratory phase IIB randomised, placebo-controlled trial. eClinicalMedicine.

[37] Hurt C.N., Jones S.E.F., Madden T.-A., Fiander A., Nordin A.J., Naik R., Powell N., Carucci M., Tristram A. (2018). Recurrence of vulval intraepithelial neoplasia following treatment with cidofovir or imiquimod: Results from a multicentre, randomised, phase II trial (RT3VIN). BJOG.

[38] Pepas L., Kaushik S., Nordin A., Bryant A., Lawrie T.A. (2015). Medical interventions for high-grade vulval intraepithelial neoplasia. Cochrane Database Syst. Rev..

[39] Cokan A., Pakiž M., Serdinšek T., Dovnik A., Kodrič T., Repše Fokter A., Kavalar R., But I. (2021). Comparison of conservative treatment of cervical intraepithelial lesions with imiquimod with standard excisional technique using LLETZ: A randomized controlled trial. J. Clin. Med..

[40] Huh W., Asirwa F.C., Hull L., Smith S., Cantu-De León D.F., Warsi Q.A., Chien E., Cox J. (2026). Phase 1b/2a randomized, double-blind study demonstrates potential of ABI-2280, a novel antiviral vaginal insert for the treatment of persistent high-risk/oncogenic HPV infection. Gynecol. Oncol..

[41] A Study of ABI-2280 Vaginal Insert (Previously Referred to as ABI-2280 Vaginal Tablet) in Participants with Cervical Intraepithelial Neoplasia. ClinicalTrials.gov Identifier NCT05502367. NCT05502367.

[42] A Randomized, Placebo-Controlled Study to Evaluate Clearance of High-Risk Human Papillomavirus and Safety After Administration of ABI-2280 Vaginal Inserts. ClinicalTrials.gov Identifier NCT06491446. NCT06491446.

[43] Antiva Biosciences Data from the Phase 1b/2 Study of ABI-2280 for Treatment of Cervical High-Risk HPV Infection. Proceedings of the Society of Gynecologic Oncology Annual Meeting on Women’s Cancer.

[44] Tang Y., Tong Q., Dai N., Xu C. (2023). Evaluation of the clinical efficacy of vaginal treatment options for persistent high-risk human papillomavirus infection after excisional treatment of cervical high-grade squamous intraepithelial lesions: A systematic review and Bayesian network meta-analysis. Virol. J..

[45] Tatti S., Swinehart J.M., Thielert C., Tawfik H., Mescheder A., Beutner K.R. (2008). Sinecatechins, a defined green tea extract, in the treatment of external anogenital warts: A randomized controlled trial. Obstet. Gynecol..

[46] Gross G., Meyer K.G., Pres H., Thielert C., Tawfik H., Mescheder A. (2007). A randomized, double-blind, four-arm parallel-group, placebo-controlled phase II/III study to investigate the clinical efficacy of two galenic formulations of Polyphenon E in the treatment of external genital warts. J. Eur. Acad. Dermatol. Venereol..

[47] Yap J., Slade D., Goddard H., Dawson C., Ganesan R., Velangi S., Sahu B., Kaur B., Hughes A., Luesley D. (2021). Sinecatechins ointment as a potential novel treatment for usual type vulval intraepithelial neoplasia: A single-centre double-blind randomised control study. BJOG.

[48] Hammer A., de Koning M.N.C., Blaakaer J., Steiniche T., Doorbar J., Griffin H., Mejlgaard E., Svanholm H., Quint W.G.V., Gravitt P.E. (2019). Whole tissue cervical mapping of HPV infection: Molecular evidence for focal latent HPV infection in humans. Papillomavirus Res..

[49] Xiao J., Chen Z., Xiao Y., Sui L., Wang C., Cong Q. (2023). Vulvar squamous intraepithelial neoplasia epithelial thickness in hairy and non-hairy sites: A single center experience from China. Front. Oncol..

[50] Lai B.E., Xie Y.Q., Lavine M.L., Szeri A.J., Owen D.H., Katz D.F. (2008). Dilution of microbicide gels with vaginal fluid and semen simulants: Effect on rheological properties and coating flow. J. Pharm. Sci..

[51] Stockfleth E., Beti H., Orasan R., Grigorian F., Mescheder A., Tawfik H., Thielert C. (2008). Topical Polyphenon E in the treatment of external genital and perianal warts: A randomized controlled trial. Br. J. Dermatol..

[52] Cokan A., Pakiž M. (2022). A case report and literature review of delayed telogen effluvium after topical imiquimod use in treating a cervical high-grade squamous intraepithelial lesion. Eur. J. Gynaecol. Oncol..

[53] O’Hanlon D.E., Come R.A., Moench T.R. (2019). Vaginal pH measured in vivo: Lactobacilli determine pH and lactic acid concentration. BMC Microbiol..

[54] Owen D.H., Katz D.F. (1999). A vaginal fluid simulant. Contraception.

[55] Falavigna M., Pattacini M., Wibel R., Sonvico F., Škalko-Basnet N., Flaten G.E. (2020). The Vaginal-PVPA: A vaginal mucosa-mimicking in vitro permeation tool for evaluation of mucoadhesive formulations. Pharmaceutics.

[56] Ozbun M.A., Patterson N.A. (2014). Using organotypic (raft) epithelial tissue cultures for the biosynthesis and isolation of infectious human papillomaviruses. Curr. Protoc. Microbiol..

[57] McLaughlin-Drubin M.E., Meyers C. (2005). Propagation of infectious, high-risk HPV in organotypic raft culture. Methods Mol. Med..

[58] McLaughlin-Drubin M.E., Christensen N.D., Meyers C. (2004). Propagation, infection, and neutralization of authentic HPV16 virus. Virology.

[59] Jackson R., Maarsingh J.D., Herbst-Kralovetz M.M., Van Doorslaer K. (2020). 3D oral and cervical tissue models for studying papillomavirus host-pathogen interactions. Curr. Protoc. Microbiol..

[60] Spurgeon M.E., Lambert P.F. (2019). A novel in vivo infection model to study papillomavirus-mediated disease of the female reproductive tract. mBio.

[61] A Safety and Efficacy Study of Transcription Activator-like Effector Nucleases and Clustered Regularly Interspaced Short Palindromic Repeat/Cas9 in the Treatment of HPV-Related Cervical Intraepithelial Neoplasia I. ClinicalTrials.gov Identifier NCT03057912. NCT03057912.

[62] Ravel J., Gajer P., Abdo Z., Schneider G.M., Koenig S.S.K., McCulle S.L., Karlebach S., Gorle R., Russell J., Tacket C.O. (2011). Vaginal microbiome of reproductive-age women. Proc. Natl. Acad. Sci. USA.

[63] France M., Alizadeh M., Brown S., Ma B., Ravel J. (2022). Towards a deeper understanding of the vaginal microbiota. Nat. Microbiol..

[64] Shannon B., Yi T.J., Perusini S., Gajer P., Ma B., Humphrys M.S., Thomas-Pavanel J., Chieza L., Janakiram P., Saunders M. (2017). Association of HPV infection and clearance with cervicovaginal immunology and the vaginal microbiota. Mucosal Immunol..

