# Supplementary material for: From Active Compound to Target Epithelium: Local Delivery, Preclinical Model Validity, and Clinical Endpoint Interpretation in Topical Therapy for HPV-Associated Lower Genital Tract Disease—A Narrative Review
Source: J Clin Med. 2026 Oct 2;15(19):7631. doi: 10.3390/jcm15197631 (PMC13666372; doi:10.3390/jcm15197631)
Supplement: Supplementary file 1 [file jcm-15-07631-s001.zip › jcm-4562914-supplementary.pdf]

Supplementary Material

From Active Compound to Target Epithelium: Local Delivery, Preclinical Model Validity, and Clinical Endpoint Interpretation in Topical Therapy for HPV-Associated Lower Genital Tract Disease—A Narrative Review

Table S1. Framework for appraising published studies and specifying new ones in topical therapy for HPV-associated disease of the female lower genital tract. The eight elements and their labels are those of Figure 2 and Section 10 of the main article. Under element 7 the table also covers prespecified stratification, which Section 10 addresses in the same paragraph as endpoint and follow-up. Section and table cross-references, and reference numbers in square brackets, are to the main article.

Chain element	When reading a published study	When designing a study
1. Clinical intention	For which intention was the product developed, and does the trial test that intention rather than a different one?	Define the clinical intention at the outset: prevention of acquisition, treatment of established disease, or reduction of recurrence after excision or ablation (Section 10).
2. Disease phenotype	Is the phenotype defined, and does the classification system distinguish HPV-associated from HPV-independent lesions?	Specify the phenotype and the classification system applied (Section 4). Prior treatment is handled as a prespecified stratification variable under element 7.
3. Mechanism and target	Is the biological target stated, and is it the population on which the proposed mechanism acts?	Derive the target population from the proposed mechanism, and define its anatomical location within the phenotype being treated (Section 4, Figure 1).
4. Required exposure	Could the formulation plausibly have delivered the compound to that population? Is anything reported that would allow this to be judged?	Report composition; where feasible report residence, dilution behavior, and stability in cervicovaginal conditions. Establishing a tissue exposure target would be more useful still, and none was identified for the candidates reviewed [30]. Approaches to exposure measurement, their applicability by subsite, and the minimum reporting set are set out in Section 5.1 and Table 3.
5. Preclinical model	Does the cited preclinical evidence come from a system in which the claimed mechanism could operate?	Select the model against the mechanism rather than by convention; state which question the model is being used to answer. Section 6 and Table 4 set out which question each model class can and cannot answer.
6. Comparator	Was natural history controlled, by randomization or by a design that accounts for background rates in the population enrolled?	Vehicle where withholding active treatment is acceptable and the vehicle is characterized; active surveillance for appropriately selected women with CIN 2, in whom it is an accepted management strategy [3]; and standard of care where treatment should not be deferred. Active surveillance does not extend to CIN 3, to unselected high-grade lesions, or to settings in which invasion is suspected.
7. Endpoint and follow-up	Is the endpoint appropriate to the phenotype, assessed over a sufficient interval, and does it correspond to what the authors conclude? Are divergent endpoints reported, or resolved selectively (Section 7)? Were age, prior treatment, and immune status accounted for?	Match the endpoint to phenotype and intention. The appropriate hierarchy differs between prevention, primary treatment, and recurrence prevention; where both histological and virological outcomes are measured, prespecify them according to clinical intention and report discordance between them transparently. Assess regression at time points that permit meaningful comparison with the natural-history trajectory of the relevant phenotype (Section 7). Extend follow-up sufficiently beyond treatment to capture clinically meaningful recurrence; longer-term assessment matters particularly for recurrence endpoints. Prespecify stratification; age, immune status, and prior treatment are among the clinical covariates most

Chain element	When reading a published study	When designing a study
		consistently relevant to the populations studied [4,6,12,13,17].
8. Inference	Does the evidence support the mechanistic claim, or an observation of change?	State in advance which inference the design can support, and which it cannot.

Note. Published studies may address some elements of the framework and not others. The framework identifies which conclusions are supported and which require additional work; it does not classify studies as valid or invalid. It concerns translational interpretability and is intended to complement, not replace, conventional assessment of internal validity, including randomization, allocation concealment, masking, sample size, adherence, attrition, and outcome ascertainment. This framework is conceptual and author-derived. It has not undergone formal validation as a risk-of-bias or quality-assessment instrument, and is not offered as an alternative to established appraisal tools.

Abbreviations: CIN, cervical intraepithelial neoplasia; HPV, human papillomavirus.
